# Potential Involvement of Adiponectin Signaling in Regulating Physical Exercise-Elicited Hippocampal Neurogenesis and Dendritic Morphology in Stressed Mice

**DOI:** 10.3389/fncel.2020.00189

**Published:** 2020-07-03

**Authors:** Pingjie Wang, Yiyao Liang, Kai Chen, Suk-Yu Yau, Xin Sun, Kenneth King-Yip Cheng, Aimin Xu, Kwok-Fai So, Ang Li

**Affiliations:** ^1^Guangdong-Hong Kong-Macau Institute of CNS Regeneration, Joint International Research Laboratory of CNS Regeneration Ministry of Education, Jinan University, Guangzhou, China; ^2^Department of Rehabilitation Sciences, The Hong Kong Polytechnic University, Kowloon, Hong Kong; ^3^Department of Health Technology and Informatics, The Hong Kong Polytechnic University, Kowloon, Hong Kong; ^4^Department of Medicine, Li Ka Shing Faculty of Medicine, The University of Hong Kong, Pokfulam, Hong Kong; ^5^Department of Pharmacy and Pharmacology, Li Ka Shing Faculty of Medicine, The University of Hong Kong, Pokfulam, Hong Kong; ^6^State Key Laboratory of Pharmaceutical Biotechnology, The University of Hong Kong, Pokfulam, Hong Kong; ^7^State Key Laboratory of Brain and Cognitive Sciences, The University of Hong Kong, Pokfulam, Hong Kong; ^8^Department of Ophthalmology, Li Ka Shing Faculty of Medicine, The University of Hong Kong, Pokfulam, Hong Kong; ^9^Guangzhou Regenerative Medicine and Health Guangdong Laboratory, Guangzhou, China; ^10^Co-innovation Center of Neuroregeneration, Nantong University, Nantong, China

**Keywords:** depression, voluntary exercise, adiponectin, dentate gyrus, hippocampal neurogenesis, dendritic plasticity

## Abstract

Adiponectin, a cytokine secreted by mature adipocytes, proves to be neuroprotective. We have previously reported that running triggers adiponectin up-regulation which subsequently promotes generation of hippocampal neurons and thereby alleviates depression-like behaviors in non-stressed mice. However, under the stressing condition, whether adiponectin could still exert antidepressant-like effects following exercise remained unexplored. In this study, by means of repeated corticosterone injections to mimic stress insult and voluntary wheel running as physical exercise intervention, we examined whether exercise-elicited antidepressive effects might involve adiponectin’s regulation on hippocampal neurogenesis and dendritic plasticity in stressed mice. Here we show that repeated injections of corticosterone inhibited hippocampal neurogenesis and impaired dendritic morphology of neurons in the dentate gyrus of both wild-type and adiponectin-knockout mice comparably, which subsequently evoked depression-like behaviors. Voluntary wheel running attenuated corticosterone-suppressed neurogenesis and enhanced dendritic plasticity in the hippocampus, ultimately reducing depression-like behaviors in wild-type, but not adiponectin-knockout mice. We further demonstrate that such proneurogenic effects were potentially achieved through activation of the AMP-dependent kinase (AMPK) pathway. Our study provides the first evidence that adiponectin signaling is essential for physical exercise-triggered effects on stress-elicited depression by retaining the normal proliferation of neural progenitors and dendritic morphology of neurons in the hippocampal dentate gyrus, which may depend on activation of the AMPK pathway.

## Introduction

The main symptoms of depression are lack of pleasure, fatigue, irritability and inattention, together with impairments in sleep, appetite and cognition, and even suicidal tendencies (Nestler et al., 2002). Studies have shown that depression and depressive symptoms are among the leading health care costs in China with about 14.7% of personal medical expenditure (Hsieh and Qin, 2018). According to World Health Organization, severe depression will become the major cause of disability in 2030 (Mathers et al., 2007). Currently, there are many hypotheses concerning the etiology of depression, including dysfunction of 5-hydroxytryptamine, noradrenergic, dopaminergic and glutamatergic systems, increased inflammation, hypothalamic-pituitary-adrenal (HPA) axis abnormality, vascular changes, and impairment on neurogenesis and neuroplasticity (Dean and Keshavan, 2017).

Adiponectin (ADN) is one of the most abundant cytokines secreted by mature adipocytes into the blood, accounting for about 0.01% of the total plasma proteins (Li et al., 2015). ADN that contains 244 amino acids with a NH_2_-terminal collagen domain and a COOH-terminal spherical domain belongs to the complement 1q family, and has a highly similar homology among different species (Bloemer et al., 2018). ADN barely exists in the form of monomers, but aggregates into a variety of oligomeric complexes, including trimers, hexamers, and high molecular weight polymers containing more than 12 monomers. Globular ADN is a derivative of the monomer following the enzymatic digestion by leucocyte elastases. Two well-studied functional ADN receptors, AdipoR1 and AdipoR2, show distinct affinities to different oligomeric complexes: AdipoR1 has a high affinity to the globular form, while AdipoR2 has comparable affinities to both globular and full-length forms (Li et al., 2015). It has been demonstrated that ADN signaling participates in the regulation of numerous biological processes throughout the body, such as enhancing insulin sensitivity, modulating inflammation and energy expenditure (Lee and Kwak, 2014; Li et al., 2015), alleviating hypertensive vascular injury and protecting cardiovascular function (Guo et al., 2018), reducing oxidative stress and ameliorating renal endothelial dysfunction (Zha et al., 2017), promoting liver glucose and lipid metabolism (Holland et al., 2017), etc. Recent studies have revealed that ADN can enter the central nervous system through the blood-brain barrier, regulating nerve activity and neural cell generation. In the hippocampus, ADN promotes neurogenesis through AdipoR1 (Yau et al., 2014a) and affects synaptic function through AdipoR2 (Zhang et al., 2017). Adiponectin can induce long-term chemical enhancement and promote presynaptic release probability (Pousti et al., 2018).

Neurogenesis refers to the process of producing new neurons from pluripotent stem cells. Currently, it is putative that neurogenesis also occurs in the subgranular zone of the hippocampal dentate gyrus (DG) in adult mammals. Activated neural stem cells produce neuronal progenitor cells (NPCs) which proliferate and migrate into granular cell layer of DG, and then differentiate into neurons, astrocytes or oligodendrocytes. Enhancement of hippocampal neurogenesis that improves cognitive functions can be fulfilled by enriched environment, learning and motor training (Ryan and Nolan, 2016). Hippocampal neurogenesis and related cognitive performances are regulated by a variety of factors, such as physical exercise, inflammation and stress (Hueston et al., 2017). Exercise is able to attenuate inflammation and promote hippocampal neurogenesis (Ryan and Nolan, 2016). Early studies have shown that exercise improves spatial learning and memory by increasing hippocampal neurogenesis while reducing glucocorticoid levels, ultimately counteracting stress (Li et al., 2013a). Additionally, exercise enhances dendritic plasticity to alleviate depressive behaviors (Yau et al., 2011). As a feasible non-medicinal treatment for depression, exercise elicits therapeutic benefits lasting longer than that of routine antidepressants (Rimer et al., 2012). We have reported that Baduanjin Qigong can increase the level of plasma ADN and alleviate the symptoms of anxiety and depression in female patients with chronic fatigue syndrome (Chan et al., 2017). A previous study by us also demonstrated that without overt stress stimulation, wheel running could effectively raise ADN levels in the hippocampus of wild-type mice, promote the proliferation of hippocampal precursor cells and ultimately mitigate depression (Yau et al., 2014a). Nevertheless, whether the above-mentioned phenomena could be reproduced under the stressing condition and the corresponding involvement of ADN signaling have not yet been investigated.

Here we examined, (1) whether stress could induce depressive phenotypes in experimental mice, and affect hippocampal neurogenesis and dendritic morphology of neurons in the DG; (2) whether exercise could reduce the above-mentioned behavioral and biological changes after stress insult, and whether such effects of exercise require the participation of ADN signaling. Elucidating the role of ADN in exercise-mediated antidepressive action under stress would provide new insights for finding biomarkers that objectively indicate the severity of depression and help formulate the individualized exercise plan for neuropsychiatric diseases involving compromised hippocampal neurogenesis and dendritic plasticity.

## Materials and Methods

### Experimental Animal

All experimental animals were guided and approved by the Animal Ethics Committee of Jinan University. Two-month-old male wild-type C57BL/6J (WT) mice purchased from Guangdong Province Medical Animal Experimental Center and global adiponectin-knockout (KO) mice (established by Ma et al., 2002) of the same genetic background, sex and age raised in the Jinan University Animal Center were used and randomly assigned into different trials. The mice with free access to water and food were housed under light/dark cycle (Light: 8:00-20:00; Dark: 20:00-8:00) in the Specific Pathogen Free Animal Facility at 24°C and 60% humidity. Three major parameters explored in the current study were ADN signaling, physical exercise and environmental stress. Hence, in order to set up a comprehensive trial and avoid the unnecessary repetition of our previous study (Yau et al., 2014a), six groups of two mouse strains (WT and ADN-KO), two training types (Running and Non-running) and two pharmacological treatments (VEH or COR) were set for most of the phenotypical experiments, as listed in Table 1.

**TABLE 1:** Animal grouping for the *in vivo* experiments.

Strain	Agent (s.c.)	Exercise	Group
Wild-type (WT)	Vehicle	Non-run	WVN
	Corticosterone	Non-run	WCN
	Corticosterone	Run	WCR

Adiponectin-knockout (KO)	Vehicle	Non-run	KVN
	Corticosterone	Non-run	KCN
	Corticosterone	Run	KCR

### Corticosterone Administration

Repeated corticosterone injections have been widely used to establish the mouse model of depression (Zhao et al., 2008). Following the similar protocol as previously described (Lopes et al., 2018; Chaves et al., 2019), corticosterone (Sigma-Aldrich, St Louis, MO, United States) was dissolved with dimethylsulfoxide (DMSO, 0.1%; Sigma-Aldrich) and Tween-80 (0.1%; Sigma-Aldrich) in the sterile phosphate-buffered saline (PBS), followed by the 10-min vortexing and 1-h ultra-sonication (Branson Ultrasonics, Danbury, CT, United States) before injection. Corticosterone at a dose of 20 mg/kg body weight was subcutaneously administered daily for 3 weeks. For the control group, mice were injected of the same volume of vehicle PBS containing 0.1% DMSO and 0.1% Tween-80.

### Exercise Paradigm

**FIGURE 1:**
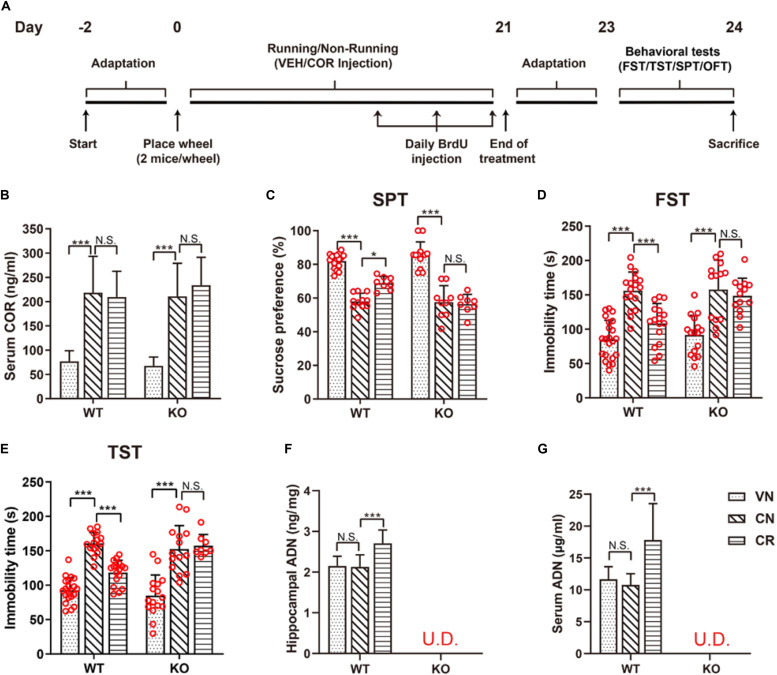
Voluntary exercise is insufficient to reduce depression-like behaviors in ADN-KO mice under stress. **(A)** Experimental timeline for animal treatments. **(B)** Changes of the serum COR level following administration of COR with or without running. *n* = 10–11 mice/group. **(C–E)** Assessments of behavioral despairs after different treatments. There was no main difference of genotype in COR-induced depressive behaviors. However, voluntary exercise substantially raised sucrose preference in the SPT (**C**; *n* = 8–18 mice/group), decreased immobility time in the FST (**D**; *n* = 13–22 mice/group) and the TST (**E**; *n* = 8–21 mice/group) in COR-treated WT mice; these effects were diminished in ADN-deficient mice. **(F,G)** COR injections did not affect the hippocampal (**F**; *n* = 8 mice/group) or serum ADN level (**G**; *n* = 8 mice/group) in WT non-runners, while the 3-week voluntary running raised both. ADN was undetected in KO mice with the designated treatments. Data are shown as means ± SD. *p < 0.05, ***p < 0.001 by two-way ANOVA and Tukey’s *post hoc* test. N.S., non-significant; U.D., undetected; FST, forced swim test; TST, tail suspension test; SPT, sucrose preference test; OFT, open field test; COR, corticosterone; ADN, adiponectin.

Voluntary wheel running was applied in this experiment as the exercise training approach (Yau et al., 2014a), which was carried out for 3 weeks concurrent with the corticosterone injections (Figure 1A). Different groups of mice were housed in the identical environment, including cages and running wheels of the same size [cage: 30 cm (height) × 30 cm (length) × 20 cm (width); wheel: 10 cm (diameter)], and in-pair housing to prevent the isolation-induced stress. The only difference, with regard to the housing environment, between the mice randomly allocated into the running group and those into the non-running group was that wheels for the non-running group were continuously locked, whereas wheels for the running group were unlocked after the 2-day adaptation period before the onset of treatments (Figure 1A). The total distance that the running wheel rotated was recorded and calculated by the Activity Wheel Monitor Software (Lafayette Instrument Company, Lafayette, IN, United States). The mean daily running distance measured in the pilot experiment was comparable between a KO mouse (4.51 ± 0.24 km, *n* = 18) and a WT control (4.50 ± 0.21 km, *n* = 18; *p* > 0.05 by Student’s *t*-test).

### Behavioral Tests

The open field test (OFT) was used to examine the mouse locomotor function (Walsh and Cummins, 1976). Briefly, mice were placed in a 50 cm (high) × 50 cm (length) × 50 cm (width) plastic box. To minimize the unnecessary anxiogenic stimulation, OFT was carried out under the noise-free and low-illuminating condition. In addition to the 2-day adaptation in the behavioral facility (Figure 1A), mice were again allowed to get accustomed to the above-mentioned environmental condition for 2 h and the transferring of the mice between the cage and the arena was exclusively performed by use of a transport compartment (a box) to reduce the handling stress. Also, after each test, the residual odor was removed, and the tested mice were placed in transition cages. The mouse trajectory was recorded for 10 min with the EthoVision software (Noldus Information Technology, Wageningen, Holland). Parameters related to the locomotor function were analyzed, including the total moving distance and the mean velocity. The sucrose preference test (SPT) was performed as previously reported (Yau et al., 2014a). Two bottles with the one containing water and the other sucrose solution (2%) were provided. After 12 h, the positions of the bottles were exchanged. Bottles were weighed before and after the experiment to calculate the net consumption of sucrose solution and water. The index “Sucrose Preference (%)” equaled to the percentage of the weight of sucrose solution consumed over the total weight of liquids consumed (i.e., sucrose solution plus tap water), which is widely employed to reflect anhedonia and inversely associated with the severity of depression. The forced swim test (FST) was another behavioral test frequently used to measure the mouse depression-like severity (Can et al., 2012). In brief, the mouse was placed in a water cylinder and the water temperature was kept at 25°C. A camera was used to record mouse swimming trajectory for 6 min, and the last 4 min was collected for scoring the immobility time. Mobility was defined as any movement beyond what was indispensable for keeping the head above the water surface. The tail suspension test (TST) was performed as described before (Steru et al., 1985). The mouse was suspended by hanging up the tail connected with a nylon rope. A session that lasted for 6 min was video-taped and analyzed for the immobility time. Slight movements confined to the front limbs and momentum-elicited swings following earlier struggles were not regarded as mobility. All the above tests were performed and analyzed in a double-blind fashion. It is worth noting that in compliance with the approved ethic guideline to reduce excessive stress to animals, and also to avoid the influence of one behavioral test on another, each mouse in the current study was only subjected to one kind of behavioral test before sacrifice (Figure 1A).

### Blood Collection

To avoid the potential circadian fluctuation of circulating levels of cytokines and to obtain the enough amount of serum for multiple ELISAs, the blood collection for all mice was performed at the fixed time point (between 14:00 and 16:00) using our long-adopted protocol. In brief, 24 h after the cessation of experiments, mice were deeply anesthetized with sodium pentobarbital (150 mg/kg body weight; Alfasan International BV, Woerden, Holland) prior to exposing the heart. About 300 μl of the whole blood from each animal were obtained by the left ventricle paracentesis using a sterile syringe (BD Corporation, Franklin Lakes, NJ, United States), and immediately transferred into a tube. Blood samples were allowed to clot at room temperature for 30 min, followed by centrifugation (1500 × *g*) for 10 min at 4°C. Aliquots of the supernatant serum were stored at −80°C for later use.

### RNA Extraction, Reverse Transcription and Quantitative Real-Time PCR

Dynamic changes of target genes were identified by the real-time PCR detection of their mRNA transcripts. In brief, after deeply anesthetized by intraperitoneal injection of sodium pentobarbital and subsequent blood collection as mentioned above, the mouse was immediately perfused with ice-cold PBS to wash away the residual blood in brain tissues. Thereafter, the mouse brain was quickly exposed with bilateral hippocampi harvested under a dissection microscope. Following the manufacturer’s protocol, each hippocampus was lysed with 1 ml of the TRIzol (Invitrogen, Carlsbad, CA, United States) using a hand-hold homogenizer (Thermo Fischer, Pittsburg, PA, United States), and placed at room temperature for 5 min prior to vigorously mixing with 0.2 ml of chloroform for 15 s. After another 2 min incubation at room temperature, the mixture was centrifuged (14000 × *g*) at 4°C for 15 min. The uppermost layer containing the total RNA was transferred to a new tube and further mixed with an equal volume of ethanol (70%). The sample was loaded onto the RNA Spin Cartridge provided in the PureLink Micro-to-Midi Total RNA Purification System (Invitrogen), and centrifuged at 12000 × *g* for 15 s, followed by sequentially washing with the Wash Buffer I (700 μl) and Wash Buffer II (500 μl for twice). The column was dried by centrifugation at 12000 × *g* for 1 min, and 50 μl of the RNase-free water was used to elute the RNA. Total RNA was further digested with DNase I (Sigma-Aldrich) to leave no trace of genomic DNA contamination. Complementary DNA (cDNA) templates were synthesized with the TaqMan Reverse Transcription Reagents (Invitrogen) using the suggested protocol. One microgram of total RNA quantified by spectrophotometry was added into a 50-μl system and reacted with the parameters below: 25°C for 10 min, 48°C for 30 min, and 95°C for 5 min. Negative controls with the reverse-transcriptase omitted from the system were set to verify the absence of genomic DNA in samples. The Taqman Gene Expression assay was carried out in quadruplicate for each cDNA sample as previously recorded (Li et al., 2010, 2012). For each well, 2 μl of the cDNA template generated in the 50-μl RT system were added into the reaction mix comprising 10 μl of the 2x PCR master mix (Premix Ex Taq kit, Takara Biotechnology Co. Ltd., Dalian, China), 1 μl of the 20x FAM labeled MGB Taqman probe (Applied Biosystems Inc., Foster City, CA, United States), and 7 μl of the nucleotide-free ultrapure water (Sigma-Aldrich). Assays were run on the MyiQ2 Two-Color Real-Time PCR Detection System (Bio-Rad Laboratories, Hercules, CA, United States) using the following ramping protocol: 95°C for 45 s, and 40 cycles of 95°C for 5 s and 60°C for 30 s. The relative expression levels of specified genes were all normalized to that of WT vehicle-injected non-runners (WVN) after 2^–ΔΔCT^ calculation, using mouse β-actin as the endogenous control. The Taqman probes are listed in Supplementary Table 1.

### Immunohistochemistry

After deeply anesthetized by intraperitoneal injection of sodium pentobarbital, mice were perfused sequentially with normal saline and 4% paraformaldehyde (PFA). Brains were fixed in 4% PFA overnight at 4°C and dehydrated with 30% sucrose until they sank. After removing the cerebellum and olfactory bulbs, the brain was coronally cut into 40-μm slices from Bregma −1.34 mm to −3.80 mm with a microtome (Thermo Fischer), and the sections in a 1-in-6 series from one animal were stored in the same well of a 24-well microplate in the cryo-protectant (30% sucrose and 30% ethylene glycol in 0.1 M phosphate buffer) at −20°C until further use, as previously described (Yau et al., 2014a). Brain sections containing the hippocampal dentate region from the same well were mounted on gelatin-coated slides (Thermo Fischer) and allowed to dry completely for 2 days. Thereafter, the samples received antigen retrieval at 99°C [for bromodeoxyuridine (BrdU) staining] or 90°C (for other targets) for 30 min in a citrate buffer (0.01 M, pH = 6.0; USB Corporation, Cleveland, OH, United States) supplemented with 0.05% Tween-20 (USB Corporation). Prior to the blocking step, endogenous peroxidases that interfere with diaminobenzidine (DAB) staining was inactivated by treating the sample with hydrogen peroxide (3%) for 5 min. The slices were blocked with PBS containing 5% bovine serum albumin (BSA) and 0.05% Tween-20 for 2 h at room temperature, and then probed with primary antibodies overnight at 4°C. To detect the incorporated BrdU, after completing antigen retrieval, the sections were immersed in hydrochloric acid (1.0 M) at 37°C for 30 min, and then neutralized with borate buffer (0.1 M, pH = 8.5) for 15 min. For staining with DAB, after the unbound primary antibody had been completely washed away, a biotinylated secondary antibody was added to the section, incubated at room temperature for 2 h, and then detected with an avidin-biotin complex system (Vector Laboratories, Burlingame, CA, United States) for 1 h and observed by DAB substrate (Invitrogen). The slides were dehydrated with ethanol and dried with toluene (Thermo Fischer). Samples were fixed with Permount medium (Thermo Fischer). For immunofluorescent staining, a fluorophore-conjugated secondary antibody was added to the slide, and the nucleus was counterstained with DAPI. The sections were fixed with a fluorescent mounting medium (DAKO, Carpinteria, CA, United States) and stored at −20°C for later analysis. Antibodies for detection included: rat anti-BrdU (1:1000; Abcam, Cambridge, MA, United States), rabbit anti-doublecortin (DCX, 1:200; Abcam), rabbit anti-Ki67 (1:1000, DAKO), biotinylated goat anti-rabbit IgG (1:200, DAKO), and goat anti-rat IgG Alexa Fluor 594 (1:200, Invitrogen). Cytogenesis in the hippocampal DG was estimated by the numbers of newborn cells, proliferating cells, and immature neurons that were positive for BrdU, Ki67, and DCX, respectively, Cell counting was performed with a semi-automatic system using StereoInvestigator software (MicroBrightfield Inc., Williston, VT, United States). With the bright or fluorescent illumination, as appropriate, the dentate region of a hemisphere was delineated under the microscope (Axioplan, Zeiss, Oberkochen, Germany) using the 4x objective. Thereafter, positively stained cells on the coronary sections were numerated under the 20x objective by use of the optical fractionator system (grid size: 55 μm × 55 μm; counting frame: 35 μm × 35 μm) following the similar protocol we have long adapted (Yau et al., 2012; Zhang et al., 2012). Six sections per mouse containing the DG were selected, whose coordinates were comparable amongst all the subjects when staining the same marker. As usual, cells located in the sub-granular zone and granular cell layer of the DG were counted, whereas those resided in the topmost focal-plane were excluded. The total cell quantity of a single section estimated by the software was further divided by the corresponding region size to get the normalized density; the average density from six slides was used to compare between different groups. For *in vivo* BrdU incorporation, the injection stock (10 mg/ml) was freshly prepared by dissolving BrdU crystal (Sigma-Aldrich) in warm physiological saline with gentle vortex. The filtration-sterilized BrdU solution was daily administered by intraperitoneal injection (50 mg/kg body weight) for the last 3 days of the training period with a 24-h interval (Figure 1A), as reported before (Yau et al., 2018).

### Golgi Staining

The morphology of granule cells in the DG was visualized with FD Rapid Golgi staining kit (FD Neuro Technologies, Columbia, MD, United States). The procedures of animal sacrifice and brain isolation were consistent with those applied for RNA extraction. Following incubation in the solution containing the equal volumes of Reagents A and B for 2 weeks at room temperature, the brain was transferred to Reagent C for another week, and then coronally sectioned into 150-μm slices using a vibratome (Leica, Wetzlar, Germany). Thereafter, the sections were soaked in the mixed solution of Reagents D and E for 10 min, sequentially dehydrated in 50, 75, 95, and 100% ethanol, cleared in xylene and mounted with resin. The granule cell morphology was imaged in a 20x objective and structured by Neurolucida software (MBF Bioscience, Williston, VT, United States). Sholl analysis was performed to quantify the number of dendritic branches and the dendritic length at the concentric 10-μm interval. Three to six neurons that were clearly visible in the DG of each animal were imaged. As described before (Yau et al., 2016), spine density was determined by randomly selecting a terminal segment of a dendritic branch longer than 10 μm for high-magnification tracing (63x objective). Three to five tertiary dendrites with at least one branch point were selected for quantification, and the visible spines along the branch segment were counted. For each neuron, ten branches were chosen for spine count. The analysis was performed in a sample-blind manner. The data were presented in units of spine numbers/10 μm.

### Western Blot

Freshly isolated hippocampal tissues were lysed with a radioimmunoprecipitation assay (RIPA) buffer (Pierce, Rockford, IL, United States) containing the inhibitors to proteinases and phosphatases (Millipore, Billerica, MA, United States), as well as phenylmethanesulfonyl fluoride (Sigma-Aldrich). Samples were homogenized and sonicated with a 50% pulse for 20 s and cleared by centrifugation (10000 × *g*) at 4°C for 30 min. The supernatant protein was collected and quantified with the BCA protein assay (Bio-Rad Laboratories). Hippocampal homogenate containing 30 μg of protein per lane was separated by SDS/PAGE and transferred to polyvinylidene fluoride membranes (Bio-Rad Laboratories). Non-specific binding was blocked with 3% BSA dissolved in Tris-HCl buffer containing 0.1% Tween-20 for 1 h. Blots were then probed overnight at 4°C with primary antibodies, followed by 1-h incubations with secondary antibodies conjugated to horseradish peroxidase (HRP), and then developed by chemiluminescence detection (Luminata Forte, Millipore). The antibodies used for detection were as follows: rabbit anti-GAP43 (1:6000; Abcam), and rabbit anti-PSD95 (1:6000), rabbit anti-Synaptophysin (1:6000), rabbit anti-SNAP25 (1:6000) and rabbit anti-β-Tubulin (1:1,000, all from Cell Signaling Technology).

### Isolation and Culture of Neural Precursor Cells

Primary neural precursor cells (NPCs) were isolated from the hippocampal DG of WT mice as previously described (Babu et al., 2011; Ryan et al., 2013). Briefly, mice were sacrificed by injecting an excess of sodium pentobarbital, and intact brains were immediately removed from the skulls and rinsed with ice-cold Neurobasal medium (Invitrogen). The hippocampal DG was segmented under a stereomicroscope, and the tissues were quickly shredded with a sterile razor blade and transferred to a 15-ml conical tube (Corning Costar, Corning, NY, United States) containing pre-warmed TrypLE reagent (5 ml; Invitrogen) and papain (10 mg; Worthington Biochemical Co., Lakewood, NJ, United States). The cells were enzymatically released for 30 min at 37°C with gentle agitations every 5 min, and then filtered through a 70-μm pore size cell strainer (BD Biosciences, San Jose, CA, United States). The obtained NPCs were maintained in a neuronal medium containing 2% B-27 supplement, 20 ng/ml fibroblast growth factor-2, 20 ng/ml epidermal growth factor and 50 μg/ml gentamicin under a humidified atmosphere containing 5% CO_2_ at 37°C. Upon subculture, neurospheres were dissociated into single cell suspensions by treatment with TrypLE reagent, and cells were counted using a hemocytometer and seeded at a density of 2 × 10^4^ cells/ml in an Ultralow Attachment flask (Corning Costar). The third to seventh passages were used in this study.

### Enzyme-Linked Immunosorbent Assay (ELISA)

In order to determine the activation of AMP-activated protein kinase (AMPK), protein kinase B (AKT), mitogen-activated protein kinase (MAPK), and extracellular signal-regulated kinase (ERK) pathways in the hippocampus, we quantified the phosphorylated proteins by ELISA. The relative amounts of phospho-Erk1/2^T202/Y204^ and phospho-Akt^S473^ were measured with the ERK1/2 (pT202/Y204) (#ab176640) and the AKT1/2/3 (pS473) (#ab176635) SimpleStep ELISA kits (both from Abcam), respectively, using the protocols recommended by the manufacturer. Hippocampal tissue proteins were prepared as mentioned above (see “Western Blot”), and 50 μg of the total protein were loaded onto each well and mixed with the Antibody Cocktail. Thereafter, the TMB substrate was added and reacted in the dark with constant shaking. The Stop Solution was supplemented to discontinue the chromogenic reaction with the optical density (OD) measured instantaneously. The OD of the sample was converted to the amount of phosphorylated protein according to the plotted standard curve, and the relative concentration was subsequently normalized to that of the WVN control. The measurement of phospho-AMPKα^T172^ was performed with the commercial ELISA kit (ab154468; Abcam) following the similar protocol above. Likewise, the semi-quantification of phospho-p38MAPK^T180/Y182^ was conducted with the PathScan Phospho-p38MAPK (Thr180/Tyr182) Sandwich ELISA kit (#7946C; Cell Signaling Technology). Assessing the phosphorylation of AMPK in NPCs was performed with a commercial ELISA kit (#KHO0651, Invitrogen) following the manufacturer’s recommendation. In brief, mono-layered WT NPCs grown on 6-well plates were incubated with the specified substances for 30 min, and rapidly lysed to extract the total protein. Lysates were quantified and 80 μg of proteins were loaded onto each well. The absorbance at 450 nm was transformed into the concentration of phosphorylated-AMPK according to the standard curve, and the relative amount was normalized to the non-treated controls that received no agent stimulation. For experiments involving combinative drug treatments, cells were pre-incubated with inhibitory agents before applying agonists.

Serum and hippocampal concentrations of cytokines including ADN, BDNF, IGF-1, VEGF, and NGF, as well as serum levels of COR were similarly determined with the below commercial ELISA kits following the recommended protocols: Mouse Adiponectin Immunoassay Kit (#32010; Antibody and Immunoassay Services, University of Hong Kong, Hong Kong SAR, China), ELISA Kits for Brain Derived Neurotrophic Factor (#SEA011Mu), Insulin-Like Growth Factor-1 (#SEA050Mu), Vascular Endothelial Growth Factor A (#SEA143Mu), Nerve Growth Factor (#SEA105Mu; all from USCN life Science; Wuhan, Hubei, China), and Corticosterone ELISA kit (#ADI-901-097; Enzo Life Sciences, Farmingdale, NY, United States).

### CyQuant Assay

To estimate the relative proliferating capacity of cells, the CyQuant assay (Invitrogen) that quantifies the nuclear DNA content was employed. Testing agents, including trimeric ADN (Antibody and Immunoassay Services), COR (Sigma-Aldrich), and Compound C (Cpd C, Millipore) were added into the serum-free media to attain the final concentrations as specified. After treatments, culture media were totally decanted and plates were immediately transferred to the −80°C deep freezer overnight. On the next day, plates were thawed at room temperature, and 200 μl of the GR CyQuant working solution were added into each well following the manufacturer’s protocol. Five minutes later, sample fluorescence (Excitation/Emission wavelengths: 480 nm/535 nm) was measured using the Victor3 Multilabel Plate Reader (Perkin Elmer, Waltham, MA, United States), and the relative cell proliferation by the CyQuant method was calculated according to the following equation: Cell Proliferation (%) = (OD_Sample_ - OD_Blank_)/(OD_Control_ - OD_Blank_) × 100%, where readings of cell-free wells were used as the blank control, and those without treatment were used as the positive control unless otherwise specified.

### Immunophenotypical Detection of the Neuronal Lineage

The subset of NPCs differentiating into the neuronal lineage was identified immunophenotypically. To be brief, at the end of Day 6, the differentiation medium was removed and cells were washed with pre-warmed PBS twice. Samples were fixed with 4% PFA for 15 min and permeabilized with 0.1% saponin for 10 min at room temperature, followed by blocking with 3% BSA for 1 h. Thereafter, coverslips were bathed into PBS containing 1% BSA and diluted primary antibodies in a humidified chamber overnight at 4°C. After being washed with PBS for three times, cells were probed with fluorophore-conjugated secondary antibodies for 1 h. DAPI was applied to counterstain nuclei. Coverslips were mounted with the Fluorescent Mounting Medium (DAKO) on slides. The antibodies used for immunodetection were: mouse anti-Tuj1 (neuronal marker; 1:1000; Covance, Berkeley, CA, United States); rabbit anti-GFAP (glial marker; 1: 1000; Millipore); goat anti-mouse IgG Alexa Fluor 568 (1:500) and goat anti-rabbit IgG Alexa Fluor 488 (1:200; both from Invitrogen). To evaluate *in vitro* neuronal differentiation, 3 independent experiments were conducted with passage-matched WT and KO NPCs derived from 3 different preparations. In each experiment, NPCs of the same genotype were seeded in parallel on 4 coverslips that were further randomly assigned into the non-treated (NT) and ADN-treated groups (i.e., *n* = 2 coverslips/group/experiment). From each coverslip, 10 representative image fields were captured randomly within the 10 pre-designated areas by a fluorescent microscope under a 20x objective (Axioplan, Zeiss, Oberkochen, Germany). Tuj1^+^ cells and DAPI-stained nuclei that helped to visualize all the cells were marked and numerated with the Image J software (NIH, Bethesda, MD, United States). The percentage of Tuj1^+^ cells on a coverslip was calculated based on the following equation: Tuj1^+^ (%) = Total number of Tuj1^+^ cells of 10 image fields/Total number of cells of the same 10 image fields × 100%. For each group, the mean and the SD were calculated from 6 individual coverslips comprising 60 image fields from three independent experiments.

### Phosphorylation Protein Array for MAPK and AKT Pathways

Cells were treated with ADN (10 μg/ml) or COR (50 μM) for 1 h. Supernatants were centrifuged (14000 × *g*, 10 min), collected and adjusted to the final concentration of 2 μg/μl total protein. Phosphorylation of MAPK pathway proteins and AKT pathway proteins were detected semi-quantitatively using Human and Mouse MAPK Pathway Phosphorylation Array and Human and Mouse AKT Pathway Phosphorylation Array C1 (RayBiotech, Peachtree Corners, GA, United States), respectively. In brief, each blotting membrane was incubated with the blocking buffer for 30 min at room temperature, and 400 μg of the protein sample were added to the membrane and probed at 4°C overnight. Thereafter, the Detection Antibody Cocktail and the HRP-Anti-Rabbit IgG reagent were sequentially added to the blot with each round of incubation for 16 h at 4°C, followed by chemiluminescence detection with a gel documentation system (ChemiTouch, Bio-Rad Laboratories) and densitomeric analyses using Image J software (NIH).

### Statistical Analysis

Data are shown as means ± standard deviations. Statistical analyses were performed with GraphPad Prism software (Version 7; San Diego, CA, United States). Student’s *t*-test was used for comparisons between 2 groups. Two-way ANOVA followed by Tukey’s *post hoc* test was employed to compare three or more sets of data, with genotype/drug or genotype/exercise as the between-subject factors; this allowed assessing the effects of COR on different genotypes or running on different genotypes under the stressing condition, respectively. For pharmacological experiments in cells, the comparisons were performed with one-way ANOVA and Tukey’s *post hoc* test. A statistical significance was set at *p* < 0.05. The “*n*” value represents the number of animals included, unless otherwise noted. Statistical analyses involving ANOVAs are tabulated in Supplementary Table 2, where the main effects tested as well as the detailed values are accessible.

## Results

### Voluntary Exercise Ameliorates COR-Induced Depressive Behaviors in an ADN-Dependent Manner

The stress-related hormone COR has been reported to cause dysfunction of the HPA axis and subsequently lead to anhedonia in rodents (Olescowicz et al., 2018). Following the specified injection protocol, blood COR concentrations of the designated groups were measured to certify the effectiveness of this strategy in raising the circulating concentration. As shown in Figure 1B, the baseline levels of serum COR in WT and KO mice were comparable (WVN: 77.0 ± 21.7 ng/ml, *n* = 11; KVN: 67.7 ± 18.0 ng/ml, *n* = 11, *p* > 0.05 for WVN vs. KVN, by two-way ANOVA; Supplementary Table 2). In contrast, daily injections of COR for 21 days substantially increased the serum COR concentration by about threefolds in non-runners of both WT and KO strains [effect of drug: *F*_(1,38)_ = 80.53, *p* < 0.0001; *p* < 0.0001 for WCN vs. WVN and for KCN vs. KVN, by two-way ANOVA; Supplementary Table 2]. The concurrent running and COR administration had neither an additive nor an inhibitive influence on the final COR levels compared with the latter manipulation alone (*p* > 0.05 for WCR vs. WCN and for KCR vs. KCN, by two-way ANOVA; Supplementary Table 2), but made the concentrations remain higher than the baselines (*p* < 0.0001 for WCR vs. WVN and for KCR vs. KVN, by Student’s *t*-test). The locomotor activity of WT and KO mice was assayed by the OFT (Supplementary Figure 1). The total travel distance and mean velocity showed that COR or physical exercise did not alter the baseline locomotor activity in WT or ADN-KO mice. There was no main difference of genotype or treatment regarding the locomotor activity (Supplementary Table 2). The anhedonic state is in line with the depressive severity, while inversely correlates with the sucrose consumption in rodents. The sucrose preference test indicated that WT and ADN-KO mice had similar percentages of sucrose consumption in non-treated condition (Figure 1C; WVN: 82.0% ± 4.3%, *n* = 17; KVN: 86.6% ± 6.9%, *n* = 13, *p* > 0.05 for WVN vs. KVN, by two-way ANOVA; Supplementary Table 2). COR administration caused comparable significant decreases in the percentage of sucrose preference in both WT and ADN-KO mice [effect of drug: *F*_(1,51)_ = 213.7, *p* < 0.0001; *p* < 0.0001 for WCN vs. WVN and for KCN vs. KVN, by two-way ANOVA; Supplementary Table 2). Interestingly, the 3-week wheel running only raised sucrose consumption in WT but not ADN-KO mice [interaction: *F*_(1,38)_ = 4.803, *p* = 0.0346; effect of genotype: *F*_(1,38)_ = 5.497, *p* = 0.0244; effect of exercise: *F*_(1,38)_ = 4.821, *p* = 0.0343; *p* = 0.0247 for WCR vs. WCN, *p* > 0.05 for KCR vs. KCN, and *p* = 0.0131 for WCR vs. KCR by two-way ANOVA; Supplementary Table 2]. Similar results were also confirmed by the forced swim test (Figure 1D), where COR increased the immobility time by almost two folds in WT and ADN-KO mice [effect of drug: *F*_(1,64)_ = 79.26, *p* < 0.0001; *p* < 0.0001 for WCN vs. WVN and *p* < 0.05 for KCN vs. KVN, by two-way ANOVA; Supplementary Table 2], and yet wheel running only shortened this duration in WT rather than ADN-KO group [interaction: *F*_(1,56)_ = 4.643, *p* = 0.0355; effect of genotype: *F*_(1,56)_ = 5.542, *p* = 0.0221; effect of exercise: *F*_(1,56)_ = 10.63, *p* = 0.0019; *p* = 0.0007 for WCR vs. WCN, *p* > 0.05 for KCR vs. KCN, and *p* = 0.0122 for WCR vs. KCR, by two-way ANOVA; Supplementary Table 2]. As well, data from the tail suspension test also echoed this observation (Figure 1E and Supplementary Table 2). The above pieces of evidence together indicate that the absence of ADN does not affect the depression-like behaviors of vehicle-treated mice. Three-week injections of COR can comparably elicit depressive phenotypes in both WT and ADN-KO mice. Nevertheless, voluntary exercise only attenuates depression-like behaviors in WT controls, suggesting that exercise-exerted antidepressive action depends on adiponectin.

### Hippocampal and Serum Levels of Adiponectin and Neurotrophins After Stress and Exercise

Based on the ELISA data, the ADN content in the hippocampus of WT mice was 2.2 ± 0.2 ng/mg tissue protein in the untreated condition (Figure 1F). COR did not change the baseline (*p* > 0.05 for WCN vs. WVN, by two-way ANOVA; Supplementary Table 2), nor did it reduce the exercise-stimulated increase in WT runners [effect of exercise: *F*_(1,38)_ = 13.56, *p* = 0.0007; *p* < 0.0001 for WCR vs. WCN, by two-way ANOVA; Supplementary Table 2; *p* < 0.0001 for WCR vs. WVN, by Student’s *t*-test]. In contrast, the hippocampal ADN remained undetectable in KO mice (below the lower detection limit “31.25 pg/ml” of the kit), despite running or COR application. The dynamic change of hippocampal ADN levels in response to the designated treatments was in agreement with that of depression-like behaviors, consequently favoring the hypothesis that ADN increase might be the enabling step for exercise to exert antidepressant-like effects. We next explored whether such increase in the hippocampal ADN content might originate from the blood circulation, as the low molecular weight ADN is known to be permeable to the blood-brain barrier. As expected, the change of ADN concentrations in the circulation coincided with that in the hippocampus (Figure 1G). The baseline serum ADN level of WT mice was 11.6 ± 2.0 μg/ml (WVN, *n* = 8), and unaffected by COR treatment under the sedate condition (WCN: 10.8 ± 1.7 μg/ml, *n* = 8, *p* > 0.05 for WCN vs. WVN, by two-way ANOVA; Supplementary Table 2). The concurrence of exercise and COR-elicited stress raised ADN concentrations to 17.8 ± 5.7 μg/ml [effect of exercise: *F*_(1,28)_ = 11.14, *p* = 0.0024; *p* = 0.0003 for WCR vs. WCN, by two-way ANOVA; Supplementary Table 2; *p* < 0.0119 for WCR vs. WVN, by Student’s *t*-test]. In contrast, neither exercise nor COR injections affected the circulating ADN levels in KO animals, which were similar among all groups and essentially undetected.

In order to verify whether the above changes potentially involved the participation of classical neurotrophic factors BDNF, IGF, VEGF, and NGF, we further measured these four targets by ELISA. The results showed that hippocampal (Supplementary Figures 2A–D and Supplementary Table 2) and serum (Supplementary Figures 2E–H and Supplementary Table 2) neurotrophins remained comparable among all groups, regardless of running and COR application. In particular, the levels did not significantly differ between WT and ADN-KO mice, largely excluding the possibility that these factors might directly lead to the above-mentioned depressive phenotypes in the current situation.

### Unaltered Expression Profile of AdipoRs and Appl1 in the Hippocampus of WT and ADN-KO Mice

It remained largely unclear whether ADN deficiency could interrupt the expression of downstream components indispensable for ADN signaling, and whether exercise and stress using the present protocol would alter their expression. Therefore, hippocampal tissues harvested directly from these trained mice were analyzed for relative levels of the designated targets. As displayed in Supplementary Figure 3, the deficiency of ADN did not change the normal expression of AdipoR1, AdipoR2, or Appl1 at the transcriptional level, as evidenced by the comparable abundance in WT and KO non-runners that received the vehicle treatment (*p* > 0.05 by two-way ANOVA, Supplementary Table 2). Neither running, nor COR-elicited stress incurred significant changes to the expression profiling of either target genes (*p* > 0.05 for WVN and KVN vs. the rest groups by two-way ANOVA; Supplementary Table 2). Based on these data, it seemed that the dynamic modulation of AdipoRs and Appl1 was less likely under the current circumstances. Thus, ADN increase following voluntary running could be the first as well as predominant factor that initiated the activation of ADN signaling in the hippocampus.

### ADN Is Indispensable for the Prevention of COR-Induced Abnormality in Hippocampal Neurogenesis by Voluntary Exercise

**FIGURE 2:**
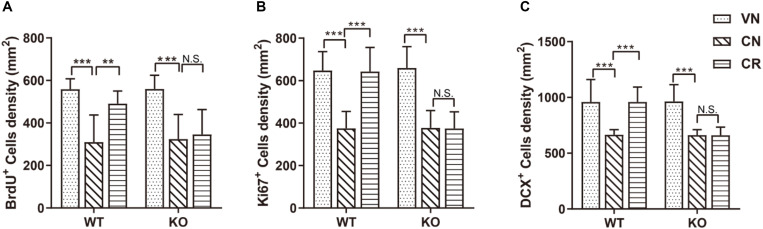
Adiponectin deficiency attenuates exercise-enhanced neurogenesis under stress. **(A–C)** COR treatment significantly lowered the number of BrdU^+^ newborn cells (**A**; *n* = 11–12 mice/group), Ki67^+^ proliferating cells (**B**; *n* = 9–15 mice/group) and DCX^+^ immature neurons (**C**; *n* = 9–15 mice/group) in both WT and ADN-KO mice. Voluntary exercise enhanced hippocampal neurogenesis by increasing all these three subsets of cells in COR-treated WT (WCR), but not ADN-null mice (KCR). Data are shown as means ± SD. **p < 0.01, ***p < 0.001 by two-way ANOVA and Tukey’s *post hoc* test. N.S., non-significant; BrdU, bromodeoxyuridine; Ki67, cell proliferation-associated nuclear antigen; DCX, doublecortin.

To investigate the underlying mechanism, we examined the hippocampal neurogenesis followed the designated treatments in both strains by performing BrdU, Ki67 and DCX immunofluorescence staining (Supplementary Figure 4). In WT mice, repeated injections of COR significantly lowered the number of BrdU^+^ cells [Figure 2A; effect of drug: *F*_(1,42)_ = 77.07, *p* < 0.0001; WVN: 559 ± 49 cells/mm^2^, *n* = 12; WCN: 310 ± 127 cells/mm^2^, *n* = 11, *p* < 0.001 for WVN vs. WCN, by two-way ANOVA; Supplementary Table 2], which was elevated by wheel running [effect of exercise: *F*_(1,42)_ = 10.18, *p* = 0.0027; *p* = 0.0013 for WCR vs. WCN, by two-way ANOVA; Supplementary Table 2]. Although ADN deficiency did not interrupt cell generation under the normal or stress condition when compared with their respective WT counterparts (*p* > 0.05 for WVN vs. KVN, and for WCN vs. KCN, by two-way ANOVA; Supplementary Table 2), it diminished exercise-exerted benefit on promoting cell generation [interaction : *F*_(1,42)_ = 6.241, *p* = 0.0165; effect of genotype: *F*_(1,42)_ = 4.268, *p* = 0.0450; effect of exercise: *F*_(1,42)_ = 10.18, *p* = 0.0027; *p* > 0.05 for KCR vs. KCN, and *p* = 0.0103 for KCR vs. WCR, by two-way ANOVA; Supplementary Table 2].

The result of Ki67 (Figure 2B) staining also confirmed the above observation. The density of Ki67^+^ population was similar in WT (WVN: 648 ± 89 cells/mm^2^, *n* = 15) and KO mice (KVN: 659 ± 101 cells/mm^2^, *n* = 13; Supplementary Table 2). COR decreased the density of Ki67^+^ cells [effect of drug: *F*_(1,42)_ = 104.3, *p* < 0.0001; *p* < 0.0001 for WCN vs. WVN, by two-way ANOVA; Supplementary Table 2], whereas running raised the value of this parameter in WT mice; such phenomenon was omitted following ADN knockout [interaction: *F*_(1,37)_ = 20.75, *p* < 0.0001; effect of genotype: *F*_(1,37)_ = 20.14, *p* < 0.0001; effect of exercise: *F*_(1,37)_ = 19.97, *p* < 0.0001; *p* < 0.001 for WCR vs. WCN, *p* > 0.05 for KCR vs. KCN, and *p* < 0.0001 for WCR vs. KCR, by two-way ANOVA; Supplementary Table 2].

Doublecortin-positive (DCX^+^) cells are newborn immature neurons. The data showed that the number of immature neurons was comparable between WT and KO mice (Figure 2C; WVN: 959 ± 202 cells/mm^2^, *n* = 15; KVN: 964 ± 151 cells/mm^2^, *n* = 14, *p* > 0.05 for WVN vs. KVN, by two-way ANOVA; Supplementary Table 2). COR significantly decreased the number of DCX^+^ cells in both WT and ADN-KO mice [effect of drug: *F*_(1,44)_ = 49.54, *p* < 0.0001; *p* < 0.0001 for WCN vs. WVN and for KCN vs. KVN, by two-way ANOVA; Supplementary Table 2]. Notably, voluntary running considerably increased the DCX^+^ cells in the COR-treated WT mice, whereas ADN-KO mice still exhibited impaired neurogenesis [interaction : *F*_(1,37)_ = 27.28, *p* < 0.0001; effect of genotype: *F*_(1,37)_ = 28.75, *p* < 0.0001; effect of exercise: *F*_(1,37)_ = 26.77, *p* < 0.0001; *p* < 0.0001 for WCR vs. WCN, *p* > 0.05 for KCR vs. KCN, and *p* < 0.0001 for WCR vs. KCR, by two-way ANOVA; Supplementary Table 2]. Collectively, voluntary wheel running prevented COR-induced suppression of neurogenesis possibly in an adiponectin-dependent way, which subsequently reduced depressive phenotypes.

### COR-Induced Impairment of Dendritic Reorganization Is Attenuated by Voluntary Exercise Through an ADN-Dependent Way

**FIGURE 3:**
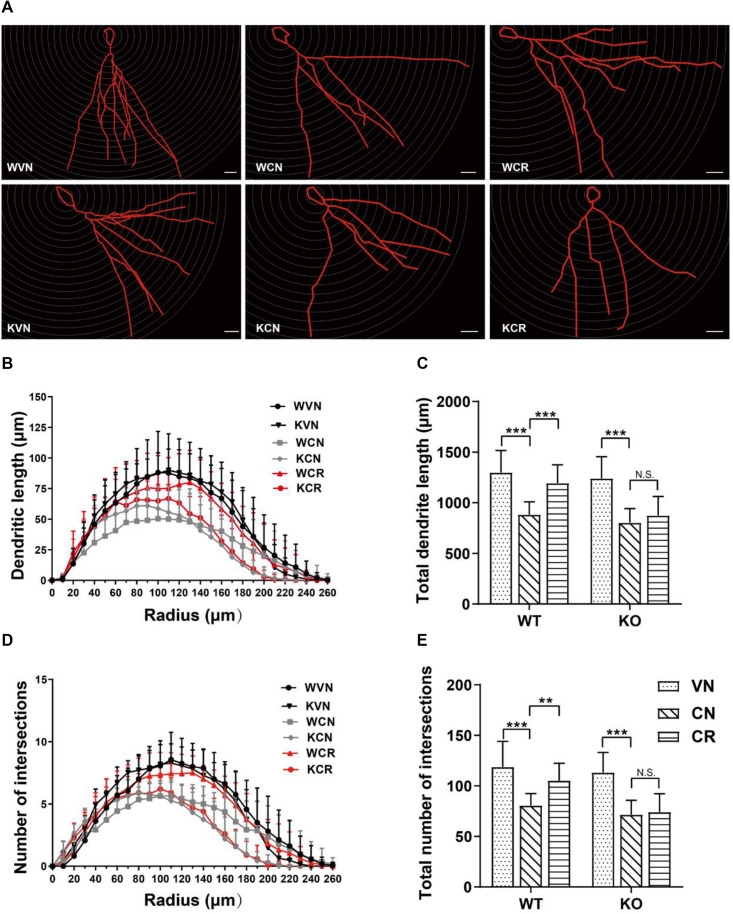
Adiponectin is required for exercise to mitigate corticosterone-elicited impairment of dendritic reorganization. **(A)** The typical morphology of neurons was reconstructed with Neurolucida software. Scale bars: 20 μm. **(B,C)** COR treatment remarkably decreased the dendritic length, as illustrated by the distribution plot of dendritic length (**B**; *n* = 12–17 neurons/group) and the average of total dendritic length (**C**; *n* = 12–17 neurons/group). **(D–E)** The number of intersections also declined after COR treatments by Sholl analysis, as evidenced by the distribution plot of the number of intersections (**D**; *n* = 12–17 neurons/group) and the average of total intersections (**E**; *n* = 12–17 neurons/group). Notably, exercise significantly increased the dendritic length and the number of intersections in WCR rather than KCR. Data are shown as means ± SD. **p < 0.01, ***p < 0.001 by two-way ANOVA and Tukey’s *post hoc* test. N.S., non-significant.

To further explore the cellular mechanism in addition to neurogenesis, we next examined whether the morphology of granule cells in the DG was changed after prolonged treatments of COR and voluntary exercise by means of Golgi staining (Figure 3A). The data displayed that the total length of neuronal dendrites (Figures 3B,C) in the hippocampal DG of WT (WVN: 1298.7 ± 218.2 μm, *n* = 16 neurons) and ADN-KO mice (KVN: 1251.4 ± 213.2 μm, *n* = 17 neurons) was equivalent before the designated treatments (Supplementary Table 2). Similarly, the total number of branch points (Figures 3D,E) was comparable between WT (WVN: 114.3 ± 18.2, *n* = 16 neurons) and KO mice (KVN: 113.2 ± 20.0, *n* = 17 neurons; Supplementary Table 2). The 21-day treatment of COR shortened the total dendritic length [effect of drug: *F*_(1,57)_ = 83.46, *p* < 0.0001; *p* < 0.0001 for WCN vs. WVN and for KCN vs. KVN, by two-way ANOVA; Supplementary Table 2], as well as decreased the number of dendritic intersections when compared with the vehicle-treated groups [effect of drug: *F*_(1,57)_ = 72.16, *p* < 0.0001; *p* < 0.0001 for WCN vs. WVN and for KCN vs. KVN, by two-way ANOVA; Supplementary Table 2]. Interestingly, voluntary exercise markedly increased the dendritic length [interaction: *F*_(1,55)_ = 6.584, *p* = 0.0130; effect of genotype: *F*_(1,55)_ = 18.50, *p* < 0.0001; effect of exercise: *F*_(1,55)_ = 16.38, *p* = 0.0002; *p* = 0.0002 for WCR vs. WCN, by two-way ANOVA; Supplementary Table 2) and dendritic intersections [interaction: *F*_(1,55)_ = 5.516, *p* = 0.0225; effect of genotype: *F*_(1,55)_ = 15.62, *p* = 0.0002; effect of exercise: *F*_(1,55)_ = 11.11, *p* = 0.0015; *p* = 0.0016 for WCR vs. WCN, by two-way ANOVA; Supplementary Table 2] in COR-treated WT mice, but not in stressed ADN-KO runners. Taken together, the results above suggested that ADN is indispensable for exercise-exerted effects on lowering COR-induced impairment of dendritic reorganization.

Dendritic spine density was calculated in the hippocampal DG region (Supplementary Figures 5A,B and Supplementary Table 2). Interestingly, neither COR nor exercise treatment affected this parameter in WT or KO mice. Additionally, semi-quantitative detections of synapse-related proteins including PSD-95, GAP43, synaptophysin and SNAP25 showed no significant change in the protein expression of either target in the hippocampal lysates of WT or KO mice after the treatments (Supplementary Figures 5C–G and Supplementary Table 2). Thus, we conclude that the antidepressant-like effect exerted by ADN may not directly rely on altering the synaptic plasticity of existing neurons.

### Voluntary Exercise Selectively Activates AMPK Signaling Pathway Through ADN

**FIGURE 4:**
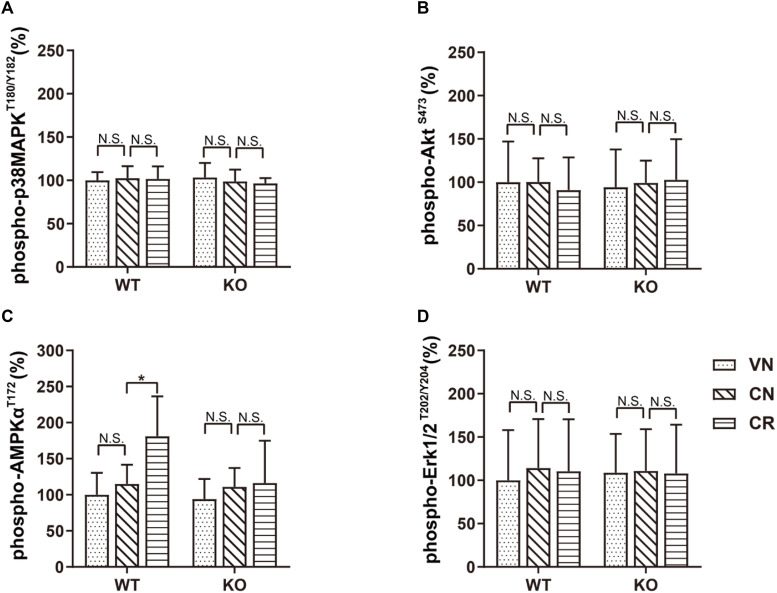
Voluntary exercise selectively activates the AMPK pathway through adiponectin. Levels of phospho-p38MAPK^T180/Y182^, phospho-AKT^S473^, phospho-AMPK^T172^, and phospho-Erk1/2^T202/Y204^ following various treatments were quantified by ELISA. **(A–D)** In both strains, COR and exercise did not affect the expression of phospho-p38MAPK^T180/Y182^ **(A)**, phospho-AKT^S473^ **(B)**, or phospho-Erk1/2^T202/Y204^ **(D)**. However, exercise significantly raised the phospho-AMPK^T172^ level in stressed WT, but not ADN-KO mice **(C)**. Data are shown as means ± SD. *n* = 10 mice/group. *p < 0.05 by two-way ANOVA and Tukey’s *post hoc* test. N.S., non-significant; MAPK, mitogen-activated protein kinase; AKT, protein kinase B; AMPK, AMP-activated protein kinase; Erk, extracellular signal-regulated kinase.

To investigate the potential pathway mediating adiponectin-elicited antidepressive effects and neurogenesis after voluntary exercise, we performed ELISA screens to examine four key molecules considered as the downstream targets of adiponectin signaling. Our data showed that treatments of COR and exercise unaffected the phosphorylation of p38MAPK, AKT and ERK (Figures 4A,B,D and Supplementary Table 2). Notably, phosphorylation of AMPK at the residue of T172 was similar in WT and KO mice (WVN: 100.0% ± 30.5%, *n* = 10; KVN: 94.2% ± 27.7%, *n* = 10, *p* > 0.05 for WVN vs. KVN, by two-way ANOVA; Supplementary Table 2). COR had no effect on the level of phospho-AMPK^T172^ in either mouse strain. The 3-week running wheel significantly upregulated phospho-AMPK^T172^ in the COR-treated WT, but not ADN-KO mice [Figure 4C; interaction: *F*_(1,36)_ = 4.685, *p* = 0.0371; effect of genotype: *F*_(1, 36)_ = 6.008, *p* = 0.0192; effect of exercise: *F*_(1,36)_ = 6.459, *p* = 0.0155; *p* = 0.0104 for WCR vs. WCN and *p* > 0.05 for KCR vs. KCN, by two-way ANOVA; Supplementary Table 2]. The results suggested that ADN is required for exercise-enhanced phosphorylation of AMPK, which is known to activate a proneurogenic pathway. In contrast, the unaltered phosphorylation of p38MAPK, AKT and ERK indicated that these three molecules, despite that they were previously reported to be activated by adiponectin in other tissues, may not get involved under the current circumstances.

### Unchanged Neuronal Differentiation in NPCs After ADN Application

**FIGURE 5:**
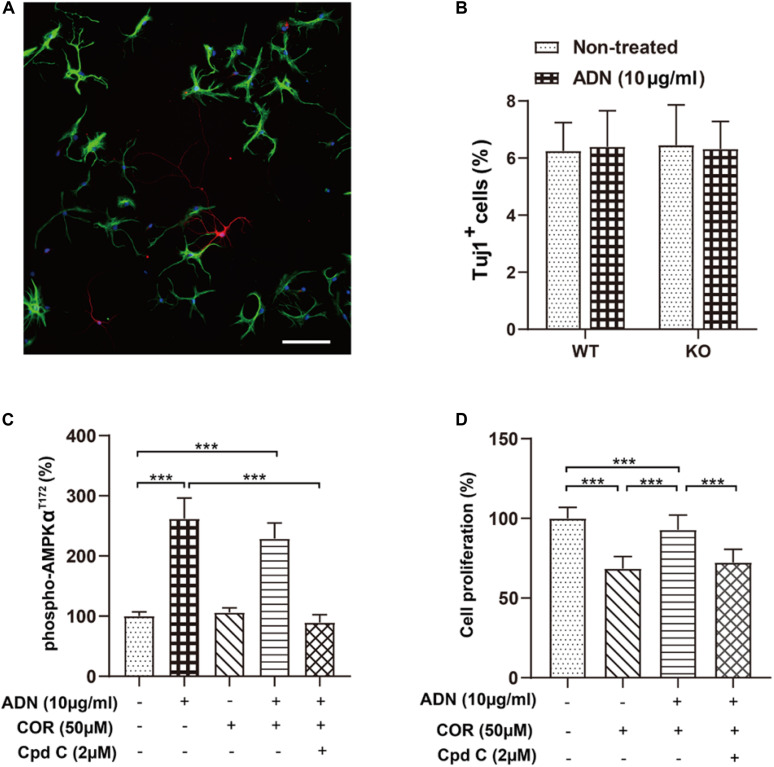
Inhibition of the AMPK pathway diminishes adiponectin-enhanced cell proliferation *in vitro.* **(A)** The representative image showing that NPCs grown on coverslips after a 6-day culture in the induction medium differentiated into the Tuj1^+^ neuronal cells (red) and the GFAP^+^ glial cells (green) with the corresponding diverse morphologies. Scale bar: 100 μm. **(B)** Adding ADN into the medium to a final concentration of 10 μg/ml did not affect the percentage of induced neuronal cells in either WT or KO NPCs; the basal neuronal differentiation was unaltered after ADN KO. *N* = 6 coverslips/group from 3 independent experiments. **(C)** The phosphorylation of AMPK at the T172 site after application of ADN, COR and Cpd C. COR did not affect the level of phospho-AMPK^T172^, whereas Cpd C essentially abolished adiponectin-induced enhancement of AMPK phosphorylation. *N* = 4 independent experiments. **(D)** Cell proliferation following ADN, COR, and Cpd C treatments. Administration of COR significantly impaired neuronal proliferation which could be reversed by adiponectin addition, whereas inhibition of AMPK phosphorylation by Cpd C potently abolished the aforementioned proliferating effect of adiponectin. *N* = 3 independent experiments. Data are shown as means ± SD. ***p < 0.001 by one-way ANOVA and Tukey’s *post hoc* test. Tuj1, Neuronal Class III β-Tubulin (neuronal marker); GFAP, glial fibrillary acidic protein (glial marker); COR, corticosterone; ADN, adiponectin; Cpd C, Compound C (AMPK antagonist).

Next, we examined whether ADN could promote newborn cells to differentiate into the neuronal lineage, in addition to its proliferative effect on NPCs. As shown in Figure 5A, 6 days after incubation with the induction medium, NPCs, either WT or KO, managed to differentiate into various neural lineages, as evidenced by the positive staining for the neuronal marker Tuj1 and the glial marker GFAP. However, the neuronal differentiation was unaffected by ADN KO (Figure 5B), given that the percentages of Tuj1^+^ population in WT (6.25% ± 0.41%) and KO NPCs (6.45% ± 0.58%) were comparable (*p* > 0.05 by one-way ANOVA; Supplementary Table 2). Moreover, application of exogenous trimeric ADN (10 μg/ml) had little influence on the percentage of Tuj1^+^ population in either WT (6.40% ± 0.51%) or KO NPCs (6.31% ± 0.40%), compared with their non-treated controls. Hence, the enhanced neurogenesis by ADN probably relies on the increase of NPC proliferation, instead of neuronal differentiation.

### Inhibition of AMPK Pathway Suppresses Adiponectin-Induced Cell Proliferation *in vitro*

Our above-mentioned results have demonstrated that voluntary exercise increased hippocampal neurogenesis and activated the AMPK pathway, both of which depend on the presence of adiponectin. To further validate whether activation the AMPK pathway directly promoted neurogenesis after ADN treatment, we determined cell proliferation *in vitro* by use of an AMPK antagonist, Compound C (Cpd C). As displayed in Figure 5C, application of Cpd C at 2 μM abolished ADN-stimulated phosphorylation of AMPK [*F*_(4,15)_ = 62.70, *p* < 0.0001; *p* < 0.0001 for COR+ADN+Cpd C group vs. ADN group, by one-way ANOVA; Supplementary Table 2]. In line with the previous *in vivo* data (Figure 4), we observed that the level of AMPK phosphorylation was unaltered after COR administration *in vitro*. Notably, administration of COR significantly impaired neuronal proliferation which could be attenuated by adiponectin addition, while inhibition of AMPK phosphorylation by Cpd C potently abolished the aforementioned proliferating effect of adiponectin [Figure 5D; *F*_(3,188)_ = 175.6, *p* < 0.0001; *p* < 0.0001 for COR+ADN+ Cpd C group vs. COR+ADN group, by one-way ANOVA; Supplementary Table 2). We further applied MAPK phosphorylation cytokine antibody arrays to screen other signaling molecules potentially involved in this process, such as CREB, JNK and mTOR, but failed to detect other candidates, because compared with the non-treated control, application of COR, ADN, or the combination of these two agents did not significantly change the phosphorylation of any targets other than AMPK (Supplementary Figure 6). Thus, the ADN-induced neurogenesis in the current situation is probably mediated through the AMPK pathway.

## Discussion

Our results show that the prolonged COR injection comparably reduced hippocampal neurogenesis and impaired dendritic morphology of DG neurons in WT and ADN-KO mice, subsequently eliciting depressive behaviors. Of note, these stress-elicited phenotypes could only be significantly reduced by voluntary wheel running in the presence of ADN. The further mechanistic investigation reveals that exercise-exerted enhancement of hippocampal neurogenesis under the stressing condition is potentially mediated through the ADN-AMPK pathway.

To explore the potential relationships among exercise, ADN as well as depression under stress, in this study, voluntary wheel running and repeated intraperitoneal injections of COR were adopted as exercise paradigm and stress insult, respectively. Wheel running is a frequently applied training mode for studying exercise and behaviors in mice (de Visser et al., 2005). Unlike other sports models such as treadmill and rotarod, it is a voluntary exercise which resembles the natural running mode of mice and can be performed under a non-stressing condition without disturbing their circadian rhythm (Goh and Ladiges, 2015; Manzanares et al., 2018). Depression models established by exogenous COR injections has been widely used (Johnson et al., 2006; Zhao et al., 2008; Liu et al., 2014; Kott et al., 2016; Wang et al., 2017). Similar to human cortisol, COR is the final product of the HPA axis activation in rodents that can satisfactorily recapitulate the impact of stress (Kott et al., 2016), where external stress causes the elevation of blood glucocorticoid levels and hyperactivity of the HPA axis, and ultimately leads to depressive symptoms (Lamers et al., 2012; Oyola and Handa, 2017). Mayer and colleagues reported that concurrent with the appearance of depressive behaviors, chronic administration of COR at 10 mg/kg for 21 days in rats significantly reduced the numbers of BrdU^+^ and DCX^+^ cells (Mayer et al., 2006). Further, treatment with COR at a high concentration (100 nM) for 20 min induced the atrophy of apical dendritic trees in the CA1 region as soon as 1 h; this could be similarly observed when incubating the slices with COR at 30 nM, but in a much delayed manner that the atrophy emerged until 2 h later. Interestingly, neither dose had an effect on basal dendrites or spine density (Alfarez et al., 2009). According to a prior study by Kannangara et al. (2011) blood corticosterone levels reflecting the stress severity were measured in different mouse groups (young or aged, individual or socially housed, sedentary or running) as designated, and they found that at the onset of active/dark cycle, running significantly reduced COR concentrations in socially housed, aged female mice as long as 4 h, concurrent with enhanced neurogenesis (Kannangara et al., 2011). In the current study, the exogenous COR application dramatically raised the circulating level of COR to >250%, which was affected by neither genotype nor exercise. Based on these, it seems that the COR injection has effectively overridden the normal COR secretion. A series of behavioral tests were next performed to probe depressive phenotypes. The data of TST and FST uniformly demonstrated that the immobility time, which parallels the depressive severity, was comparable in both mouse strains when these non-running animals were injected with vehicle. This suggests that ADN under the basal condition might be dispensable to retain the normal depressive state. Apart from the preventive effect of exercise under the basal condition we have previously reported (Yau et al., 2014a), it could also offer a similar benefit under the stressing condition, evidenced by the decrease of COR-prolonged immobility time in WT runners. Such depressive phenotypes were reassured by a third SPT that probes the core symptom of depression (i.e., anhedonia). Once again, the result was in accordance with those obtained from TST and FST, verifying that the differential depressive phenotypes between WT and KO mice could only be seen after running. Taken together, ADN-KO mice seemed devoid of the running-elicited antidepressant-like effects.

A large number of literatures have demonstrated that regular physical exercise can markedly enhance mental health and alleviate symptoms of depression, anxiety and stress (Raglin, 1990; DiLorenzo et al., 1999; Wipfli et al., 2011). Accumulating evidence supports that the benefits of physical exercise on depression are potentially fulfilled by affecting the hippocampus. For example, Gujral et al. reported that compared with medication group (12-week antidepressant treatment), exercise group (exercise + medication) displayed a more efficient trajectory of relief in depressive symptoms in both young (20–39 years’ old) and senile (60–79 years’ old) patients with major depressive disorders (Gujral et al., 2019). Likewise, a meta-analysis study on children and adolescents revealed significant effects of exercise on mental health, and confirmed that a greater amount of sedentary behavior was associated with a higher level of psychological ill-being (i.e., depression) while a lower level of psychological well-being (i.e., satisfaction with life and happiness) (Rodriguez-Ayllon et al., 2019). Physical exercise could enhance the cerebral blood volume and tissue density in the hippocampus (Maass et al., 2015; Kleemeyer et al., 2016). Besides, the abnormity of bilateral hippocampi was identified as a reliable and evident indicator for acutely depressed adults based on structural MRI research (Gujral et al., 2017). Cognitively and psychiatrically healthy senile adults were found to have larger hippocampal volumes, together with higher cardiorespiratory fitness which indicates an individual’s aerobic capacity and is frequently used as a proxy for a long-term exercise habits (Erickson et al., 2009; Szabo et al., 2011). A 12-month brisk walking of moderate intensity led to a 2% increase in the hippocampal volume (Erickson et al., 2011). These exercise-induced positive effects on mood states may depend on a variety of physiological and biochemical mechanisms, including the secretion of endorphin (Fuss et al., 2015), the regulation of mitochondrial function (Aguiar et al., 2014; Bansal and Kuhad, 2016), the activation of mammalian target of rapamycin (mTOR) (Lloyd et al., 2017), the regulation of hypothalamic–pituitary-adrenal (HPA) axis (Droste et al., 2003), and so on.

Adiponectin is an adipocyte-secreted protein molecule, whose numerous physiological functions in obesity, diabetes, inflammation, atherosclerosis and cardiovascular diseases have been extensively explored over the past two decades (Bloemer et al., 2018). As a potential marker for bipolar depression, adiponectin expression levels decreased significantly in bipolar disorder patients (Misiak et al., 2019; Platzer et al., 2019). ADN-KO mice displayed not only cognitive impairments, but also LTP deficits of the hippocampal Schaefer collateral pathway and altered levels of key presynaptic and postsynaptic proteins involved in glutamatergic neurotransmission, together suggesting that adiponectin is a crucial regulator of cognition and synaptic function (Bloemer et al., 2019). Based on prior studies, ADN may exhibit its antidepressant-like property through various mechanisms, including enhancing hippocampal neurogenesis (Yau et al., 2014a; Chan et al., 2017), mitigating neuro-inflammation (Chabry et al., 2015; Nicolas et al., 2017), and modulating the tryptophan metabolic pathway (Li et al., 2020). In this study, the quantitative ELISA data tendered evidence showing that voluntary running significantly and comparably raised serum ADN levels in both vehicle- and COR-administered mice. In contrast, COR injections had no effect on decreasing the ADN baseline. The dynamic changes of circulating ADN concentrations in WT mice were consistent with the alterations of behavioral despairs, thereby reinforcing the notion proposing ADN as the important factor mediating exercise-triggered benefits. This view was further undergirded by the constant absence of ADN in KO mice, which possibly accounted for the omission of such benefits. Notably, COR treatments insignificantly affected ADN levels; this is consistent with our above speculation that, although exerting distinct effects, COR and ADN might function through non-identical mechanisms. ADN present in the CNS has been putatively regarded to come from the circulating pool through the blood-brain barrier (Yau et al., 2014a). Based on these hints, if ADN accounts for the exercise-elicited anti-depressive action, the change of its level in the CNS should be secondary and consonant to that in the bloodstream. As anticipated, our data verified that ADN levels in the hippocampal lysates of WT mice changed in concert with that in the serum samples: running also stimulated an increase of ADN contents in the hippocampus of WT mice with COR application. Again, ADN was virtually undetected in the hippocampal tissues of KO mice. Considering that expressions of AdipoRs and Appl1 in the hippocampus were unaltered by exercise, we accordingly deduced that running-triggered ADN increase should be the enabling step initiating ADN signaling. It is noteworthy that, although many studies have reported that moderate physical exercise increases the plasma adiponectin level (Simpson and Singh, 2008; Saunders et al., 2012; Markofski et al., 2014; Inoue et al., 2017), some also showed that exercise did not alter (Simpson and Singh, 2008; Gondim et al., 2015) or even reduced this parameter (Simpson and Singh, 2008; Gondim et al., 2015; Gastebois et al., 2016). The discrepancies of these results may result from not only the exercise paradigms (frequency, pattern and intensity), but also the animal models (such as age, health status).

Neurotrophic factors have been implicated to mediate pro-neurogenic and pro-surviving effects of physical exercise. Given that pathways like AKT and ERK are capable of being modulated by both neurotrophic factors and ADN, it was accordingly interesting to examine whether there was any interaction between these two. BDNF is one of the most characterized neurotrophins, whose serum and hippocampal expression levels decrease under stress or depression (Duman and Monteggia, 2006; Bocchio-Chiavetto et al., 2010), while exercise significantly increased BDNF expression that lasted for 1–2 weeks after exercise intervention (Adlard et al., 2004; Berchtold et al., 2010; Marlatt et al., 2012). Surprisingly, we observed that BDNF levels were unaltered by neither treatment. Accorded with our current findings, Marlatt et al. (2012) demonstrated that a short duration of exercise (1 month) was ineffective to induce BDNF upregulation in aging animals. Yau et al. (2014b) also observed a reduction of BDNF levels in the rat hippocampus after 2 weeks of sustained running, accompanied by enhanced neurogenesis and synaptic plasticity. Likewise, voluntary running following chronic CORT treatment failed to affect plasma BDNF concentrations (Yau et al., 2012). Moreover, Sheikhzadeh et al. (2015) reported that neither long-term (8 weeks) nor short-term (2 weeks) exercise was sufficient to alter hippocampal BDNF levels in rats. Given that BDNF is known to express elsewhere (e.g., muscle) in addition to neural tissues and is dynamically modulated at both transcriptional and translational levels, we speculate that the different findings may be attributed to variations in non-identical experiment settings. To name a few, previous studies showed that subjects with a moderate exercise frequency had higher levels of serum BDNF than those with a high exercise frequency (Chan et al., 2008), and the vigorous habitual physical activity lowered BDNF levels (Nofuji et al., 2008), suggesting that the expression of BDNF are associated with exercise paradigms (e.g., mode, intensity, duration). It was reported that the magnitude of the exercise-induced effect on enhancing BDNF was greater in males (Szuhany et al., 2015), indicating the final outcome may also depend on animal models adopted (e.g., strain, age, gender). Hence, more studies with comparable experimental parameters, and yet better the *in vivo* biosensors for these neurotrophic factors are in great need to reach the consensus. Similarly, we did not observe remarkable changes in other neurotrophins (IGF-1, VEGF and NGF) between different groups, suggesting that there may be a functional segregation between ADN and other neurotrophic factors.

Previous mechanistic studies have demonstrated that insufficient hippocampal neurogenesis in adulthood plays a key role in the pathogenesis of depression (Malberg and Duman, 2003; Henn and Vollmayr, 2004; Sahay and Hen, 2007; Eisch and Petrik, 2012). Hu et al. (2016) have reported a significant correlation between the number of BrdU^+^ cells in the DG and the preference to sucrose. Similarly, we have also reported a negative correlation between the number of Ki67^+^ cells in the DG and the immobility time of TST and FST (Yau et al., 2014a). Furthermore, various anti-depressive treatments such as antidepressants, physical exercise as well as electro-convulsion significantly enhance neurogenesis (Hanson et al., 2011). Particularly, exercise which proves to be an effective and economical treatment for depression (Duman et al., 2008; Carek et al., 2011; Abdollahi et al., 2017; VanDerwerker et al., 2018) has also been known to enhance neurogenesis (Beauquis et al., 2010; Lee et al., 2016; Nishii et al., 2017; Leem et al., 2018; Yau et al., 2018), a process involves the generation of new neurons and neuronal connections in the sub-ventricular area of the lateral ventricle and the DG of the hippocampus (Curtis et al., 2012; Miller et al., 2013). In the present study, hippocampal cellular changes were examined by staining with three various markers BrdU, Ki67 and DCX. These three sets of data uniformly substantiated that exercise failed to trigger a significant increase of hippocampal neurogenesis in KO mice under stress. As well, ADN KO did not affect neuronal differentiation, as evidenced by the comparable percentages of Tuj1^+^ population after induction in WT and KO NPCs in *vitro*. Unlike BDNF, ADN seemed dispensable for neural development and maintenance; hence, the KO animals under either the basal or stressing condition behaved similarly to their WT counterparts in terms of the depressive state. Exercise possibly triggered the increase of circulating ADN, which subsequently contributed to the elevation in the hippocampus after its permeation through the blood-brain barrier. The higher level of ADN might promote hippocampal neurogenesis, consequently lowering depression. By contrast, KO mice deficient of this adipokine did not undergo this process, therefore exhibiting differential cellular and behavioral responses to exercise compared with their WT counterparts.

The increase of ADN in the hippocampal DG was presumed to activate the downstream signaling, some of which are proliferating or protective for neurons. To unravel the molecular mechanism by which ADN might enhance hippocampal neurogenesis under stress, the involvement of various candidate pathways were investigated. AMPK phosphorylation is known to play an important role in adiponectin signaling. For instance, ADN’s protection against ischemic injury is mainly related to AdipoR1-dependent AMPK phosphorylation (Liu et al., 2019). In addition, AMPK is also involved in neurogenesis (Jang et al., 2016). Furthermore, AMPK pathway participates in exercise-mediated antidepressant therapy (Odaira et al., 2019). In the current study, the ELISA-based screening of four cascades suggested the regulation of this process by the AMPK pathway, because the phosphorylated AMPK^T172^ that indicating its activation was increased in the hippocampal lysates in WT, but not KO runners, whereas the corresponding phosphorylated forms of other three pathways remained unchanged and seemed independent of specified treatments or genotypes *per se*. The relative cell proliferation quantified by the CyQuant assay was decreased to 70% after COR addition compared with the non-treated controls; this reduction could be largely attenuated almost back to the normal baseline by concomitantly applying ADN. Based on this result, it seemed that ADN could directly diminish COR-elicited suppression on hippocampal NPCs. Further addition of Cpd C abolished the aforementioned protective effect of ADN, substantiating that this mitigation was similarly dependent on the AMPK phosphorylation. This view was again consolidated by the evidence from ELISA quantification that COR at 50 μM did not affect the basal phosphorylation of AMPK^T172^. Nor did it significantly interrupt the corresponding increase elicited by ADN. In contrast, Cpd C inhibited the AMPK pathway activation, accompanied with the diminution of ADN-initiated restoration of the normal growth. Altogether, the experiments showed that COR was capable of exerting the anti-proliferating effect directly on hippocampal NPCs at relative high concentrations, which could be largely offset by ADN in an AMPK-dependent mechanism. The c-Jun N-terminal kinase (JNK) also belongs to the MAPK superfamily, and has recently been demonstrated to participate in the regulation of adult hippocampal neurogenesis. Mohammad et al. reported that suppressing JNK pathway activation by knocking out Jnk1 gene or applying the JNK inhibitor stimulated hippocampal neurogenesis and subsequently relieved anxiety as well as depression-like behaviors by imposing cell-autonomous control from the neurogenic niche (Mohammad et al., 2018). A previous study by Sun and colleagues showed that running could attenuate the phosphorylation of JNK (Sun et al., 2018). Interestingly, our proteomic array screen did not detect any significant change of JNK phosphorylation in the hippocampal lysates following voluntary wheel running in the current study. We speculate that this difference may be attributed to the varied paradigms applied, given that some prior studies have found that resistance training did not affect cortical or hippocampal JNK levels, whereas high-intensity treadmill running elevated the phosphorylation of JNK (Sun et al., 2017; Henrique et al., 2018).

While the importance of hippocampal neurogenesis has been putatively accepted, two issues remain frequently asked and therefore need elaboration. The first one is how a relatively small increase in hippocampal neurogenesis can ultimately affect brain function and plasticity. An interesting fact is that about 700 newborn neurons are added to the hippocampal DG of middle-aged people each day (Spalding et al., 2013), while this number is about 9000 in rats (Cameron and McKay, 2001). Apparently, the number of newborn neurons is negligible compared with the approximate 86 billion neurons in the human brain (Herculano-Houzel, 2012). Nevertheless, the daily newborn neurons in the hippocampus can still be functional important. Above all, persistent hippocampal neurogenesis may occur throughout the life span of human beings. With time elapses, the cumulative number could be fairly huge. According to a report, the newborn granule cells in the mouse DG potentially account for over 70% of the total population in just 1 year (Cameron and McKay, 2001). Second, as a major input region in the hippocampus, the DG receives not only commissural inputs from the contralateral hippocampus, cholinergic input from the septum, dopaminergic inputs from the mid brain, feedback inputs from the CA3 region, glutamatergic inputs from Mossy cells and inhibitory inputs from interneurons in the hilus, but also afferents from the lateral and medial entorhinal cortex which is the key input source of DG and closely related to novel sensory and environmental information integration (Vivar et al., 2012, 2016; Vivar and van Praag, 2013; Goncalves et al., 2016). Additionally, granule cells in the DG finally project to the CA3 region, forming an auto-associative network essential to store representations of sensory experience (Marr, 1971; Drew et al., 2013). Thus, once newborn granule cells integrate into the DG circuitry, the wide associations that they develop would magnify their significance. Third, the hallmark of DG circuits is a larger number of principal neurons and the sparseness of coding that is probably due to strong inhibitory inputs from interneurons in the DG and hilus (Li et al., 2013b; Goncalves et al., 2016). Compared with mature granule cells, the newborn ones are hyper-excitable (Aimone et al., 2006; Ge et al., 2006; Danielson et al., 2016; Anacker and Hen, 2017), and therefore may exert diverse effects on memory encoding: on one hand, they may associate similar information one another so as to serve as direct “pattern integrators”; on the other hand, they may also function as indirect “pattern separators” by inhibiting mature granule cells and facilitating sparse coding in the DG (Aimone et al., 2009; Anacker and Hen, 2017). Further, since the newborn neurons are involved in promoting the clearance of the previously established memories and preventing the interference between the new and the old memories, hippocampal neurogenesis may accordingly be required for cognitive flexibility (Wiskott et al., 2006; Appleby et al., 2011; Akers et al., 2014). Notably, influences of neurogenesis on emotion are somehow partly related to those on cognition. For instance, the impaired cognitive plasticity may interrupt the complete clearance of prior fear-associated memory engram and the discrete encoding of new safe conditions, consequently leading to persistent fear (Anacker and Hen, 2017). Based on quite a few clinical studies, numerous psychiatric disorders co-exist with impairment of cognitive flexibility, such as depression (Deveney and Deldin, 2006), post-traumatic stress disorder (Keith et al., 2015), and obsessive compulsive disorders (Chamberlain et al., 2007). Last, neurogenesis contributes to the maintenance of the HPA axis homeostasis. Thus, impairment of neurogenesis may initiate a vicious cycle of hyper-function of the HPA axis and further reduction of neurogenesis, ultimately resulting in prolonged disturbance in stress reactivity and sustained anxiety- and depressive-like behaviors (Schloesser et al., 2009; Snyder et al., 2011; Mateus-Pinheiro et al., 2013). Taking account of the reasons mentioned above, new neurons generated in the adult hippocampus should not be overlooked.

The other issue is the recent controversies over the existence of adult hippocampal neurogenesis in human beings. Some reported that recruitment of young neurons to the primate hippocampus decreased rapidly during the first years of life, and that neurogenesis in the DG discontinued, or was extremely rare, in adult humans (Sorrells et al., 2018). In contrast, others took a distinct view that healthy senile subjects without cognitive impairment, neuropsychiatric diseases or treatments displayed preserved neurogenesis; these researchers speculated that the ongoing hippocampal neurogenesis sustains human-specific cognitive function throughout life, and its decline may be linked to compromised cognitive-emotional resilience (Boldrini et al., 2018; Moreno-Jimenez et al., 2019). Lucassen et al. (2020) have emphasized that detailed parameters of experiments such as methods of tissue processing and preparation (e.g., block size, fixation duration) and animal conditions (e.g., agonal state) could affect the outcomes. As well, the clinical investigation and documentation of human subjects may strongly affect the quality of postmortem studies (Bao and Swaab, 2018). Given that the study by Sorrells et al. (2018) suffered from methodological drawbacks concerning postmortem delay, it is therefore unnecessary to abandon the long-term opinion that adult neurogenesis functionally contributing to neural plasticity and cognition across the human life span (Kempermann et al., 2018; Moreno-Jimenez et al., 2019; Lucassen et al., 2020). Indeed, besides the direct histological proof in postmortem human brains, there are also a large number of studies providing indirect evidence for adult hippocampal neurogenesis through changes in hippocampus-dependent functions, such as the regulation of mood and anxiety (Hill et al., 2015), spatial and fear learning and memory (Dupret et al., 2007; Drew and Huckleberry, 2017), and pattern separation (Clelland et al., 2009). Of note, our current data suggest that ADN elevation by exercise enhances hippocampal neurogenesis and subsequently mitigates depression-like behaviors in mice. Considering the easy translation of exercise to human subjects, this finding suggests a potential way to modulate hippocampal neurogenesis and thereby provides a chance for testifying the presence and significance of this biological process in adult humans. Nevertheless, discovering non-invasive *in vivo* markers for human neurogenesis would considerably facilitate research in this field and ultimately help to reach a consensus.

In addition to the possible neurogenesis mechanism mediating exercise-elicited antidepressive action, neurogenesis-independent mechanisms may get involved in this process, as well. Morphological abnormities of dendrites are closely related to impaired synaptic connectivity and plasticity, which result in the altered mental state and cognition (Penzes et al., 2011). It has been shown that there is a significant decrease in the volume of the hippocampus in depressed patients, with a marked reduction in dendritic spine density as well as dendritic arborization in hippocampal neurons (Bremner et al., 2000; Law et al., 2004; Drevets et al., 2008; Gong and He, 2015). Under the chronic stress condition, increases of corticosteroid secretion have been considered the major cause for hippocampus dendritic spine loss, synaptic dysfunction and depression-like behaviors in mice and rats (Wang et al., 2013). Administration of different doses of COR may incur varied effects on dendritic structures and electrophysiological properties. As an example, moderate doses of COR can enhance LTP in the DG but not the CA1 region of hippocampus, whereas high doses of COR can inhibit LTP in the CA1 region rather than the DG (Sharvit et al., 2015). Likewise, COR injections for consecutive 35 d reduced dendritic spine density in the hippocampal CA1 region and such effect was closely related to different parameters including the intensity and the duration of stress insult, genetic background and COR concentration (Conrad et al., 2017). Further, high levels of COR, by activating glucocorticoid receptors, reduced the dendritic complexity of young, developing pyramidal neurons in the CA1 subarea within hours (Alfarez et al., 2009). Likewise, exposure to stress for a prolonged time in adult rats induces the contraction of dendrites at the apex of hippocampal CA3 neurons and the loss of synapses (Kole et al., 2004). In post-partum female rats, the dendritic complexity of pyramidal neurons in the CA3 region was also lowered by COR at high concentrations (Workman et al., 2013). COR treatment reduced the number of branches of mature neurons in the DG, shortened the length of dendrites, but did not change the dendritic spine density of DG neurons (Yau et al., 2016). In contrast, treatment with antidepressants can effectively enhance dendritic spine density in the hippocampal CA1 region (Chidambaram et al., 2019). It is undoubted that abnormal alterations in dendritic plasticity of hippocampal neurons are important in the development of depressive neural circuitry (Chidambaram et al., 2019). In line with the above reports, here we found that COR significantly shortened the total length of dendrites in the DG and decreased the number of dendritic branches in both genotypes, but had no significant effect on dendritic spine density. Voluntary exercise has been known to improve neurogenesis in the rat hippocampus, and increase the dendritic complexity as well as total dendritic length of granular cells in the hippocampal DG (Redila and Christie, 2006). Our current data showed that voluntary wheel running attenuated the atrophy of dendrites in the DG of WT rather than ADN-KO mice. Collectively, these results further suggest that improvement of dendritic plasticity by exercise may also contribute to adiponectin-induced antidepressive action under the stressing condition. Moreover, some studies suggested that exercise increased the expression of synapse-related proteins (including PSD-95, GAP43, synaptophysin and SNAP25), implicating that exercise might promote synaptic plasticity (Cassilhas et al., 2012; Lin et al., 2012, 2015; Tsai et al., 2013); however, there are also disagreements. For instance, Fernandes et al. (2013) have showed that exercise training failed to affect the levels of synapsin I, synaptophysin and PSD-95 in the rat hippocampus. Likewise, adult-initiated exercise did not affect the hippocampal expression of synaptophysin or PSD-95 in rats (O’Leary et al., 2019), and high-intensity exercise had no effect on that of synapsin I or PSD-95, either (Shih et al., 2013). The above results are in line with our current observation that no significant change in the hippocampal levels of PSD-95, GAP43, synaptophysin and SNAP25 proteins in either group. Interestingly, some studies suggested that newborn granule cells which may exert a unique and beneficial effect on synaptic plasticity modification could be affected by exercise (Schmidt-Hieber et al., 2004; Ge et al., 2007), and a significant greater long-term potentiation (LTP) was observed in the DG after exercise (van Praag et al., 1999; Vasuta et al., 2007). Hence, future investigations with biochemical and electrophysiological assessments on the individual hippocampal subareas may reconcile these discrepancies.

“Exerkine” is a newly introduced concept used to define a variety of biologically-active compounds produced and released from exercise as well as non-exercise organs/tissues into the circulation, subsequently enabling the physical exercise-elicited crosstalk between peripheral and central systems in autocrinal, paracrinal and/or endocrinal manners (Safdar et al., 2016; Yu et al., 2017). Such physiological compounds comprise peptides, nucleic acids and metabolites, and the most extensively studied sources are skeletal muscles (myokines), liver (hepatokines), and adipose tissue (adipokines). While this research field remains little explored, exerkines are now classified into different subgroups, including immune-modulating factors (e.g., interleukins), neurotrophins (e.g., BDNF), other cytokines (e.g., irisin), microRNAs (e.g., miR-134) and metabolites (e.g., kynuretic acid) (Li et al., 2019). Some exerkines have been suggested to contribute, either directly or indirectly, to physical exercise-triggered benefits on cognition and mood by modulating neuroplasticity. In the current study, we demonstrate that adiponectin is a potential key exerkine involved in the anti-depressive action by exercise in the stressing condition. Nevertheless, similar molecules are emerging continuously. For instance, Moon et al. (2016) reported that a myokine called cathepsin B (CTSB) was increased in mouse gastrocnemius muscle as well as plasma after running, which subsequently upregulated BDNF and enhanced neurogenesis. Furthermore, treadmill exercise also raised the plasma levels of CTSB in Rhesus monkeys and humans, and such changes in CTSB levels were correlated to fitness and hippocampus-dependent memory function (Moon et al., 2016). As well, FNDC5, another myokine induced by exercise in a PGC1-α-dependent pathway, regulated neuroplasticity in an indirect manner that its overexpression in the liver via recombinant adenoviral vehicle and subsequent cleavage and secretion as irisin into the plasma could ultimately upregulate the expression of neuroprotective genes such as BDNF in the hippocampus (Bostrom et al., 2012; Wrann et al., 2013). In terms of the metabolites, exercise could elevate the kynurenine aminotransferase level in skeletal muscles through the PGC-1α1-PPARα/δ pathway, which then reduced the blood level of kynurenine, a compound able to permeate the blood-brain barrier and cause depression (Agudelo et al., 2014). Based on the accumulating evidence supporting the critical roles of exerkines, it seems to us that a series of well-controlled and comprehensive experiments are in great need. Expression profiling of exerkines is by no means a simple thing, because (i) varied exercise paradigms (e.g., frequency, intensity, pattern, duration) and individual characteristics (e.g., age, sex, race, health state) may trigger non-identical releases of exerkines; (ii) different exerkines have their own intrinsic and extrinsic regulations on transcription, translation, post-translational modifications and degradation, and thereby may exert various effects; (iii) some exerkines, as FNDC5 mentioned above, may fulfill their effects on brain by transforming into secondary signals (e.g., BDNF), consequently making the regulatory network more complicated; (iv) discrepant brain areas that take charge of differential functions do not have a uniform sensitivity even to the same exerkine due to the facts that the final concentration of the exerkine is greatly affected by the area *per se* (e.g., blood supply, volume, intactness of the blood-brain barrier) and the density of the receptors specific to the exerkines (e.g., AdipoR1 is highly expressed in medial prefrontal cortex, hippocampus and amygdala, whereas AdipoR2 is restricted to hippocampus and certain hypothalamic nuclei) (Liu et al., 2012). The aforementioned circumstances may account for the various outcomes of different studies. Hence, revealing the whole spectrum of exerkines, their dynamics regulated by exercise and downstream biological functions would not only help maximize the efficiency and safety of this non-medicinal intervention to a specific subject, but also shed light on designing and application of drugs resembling to endogenous exerkines. For example, resveratrol and epicatechin discovered to increase hippocampal BDNF expression and neurogenesis may serve as promising exercise-mimetics suitable for people unable to exercise (Guerrieri et al., 2017).

There are certain limitations in this study. For example, the immunohistological assessments including neurogenesis and dendritic morphology focused specifically on the hippocampal DG, whereas the quantification of expression levels of targets such as neurotrophic factors and phosphorylation of key signaling molecules used the whole hippocampal lysates due to the situation that the amount of proteins for the designated tests by ELISA and Western-blotting was fairly large and unable to be obtained from the DG of a single mouse. However, this would make our results inevitably suffer from interference caused by other sub-regions and reduce their credibility. Thus, application of the latest techniques like Single-molecule Array may be preferential when conducting similar experiments. Second, although plenty of evidence has substantiated the notion that enhancement of hippocampal neurogenesis leads to antidepressive effects of exercise, we did not directly testify the causal relationship between these two, given the difficulty in setting more groups. Indeed, application of anti-mitotic drug ara-C may not only examine this causative association, but also assist in estimating the potential contribution of neurogenesis and dendritic remodeling to ADN’s effects, which is therefore worthy of future exploration. Third, the exercise training and corticosterone injections were carried out concomitantly, so the benefits of running we observed might reflect both its preventive and therapeutic effects. Thus, to precisely evaluate the curative anti-depressive effects of ADN, the onset of exercise training may follow the appearance of depressive phenotype. Fourth, it has been reported that the plasma ADN level in ADN-haploinsufficient (ADN^+/–^) and ADN-KO (ADN^–/–^) mice is approximately 60 and 0% of that in WT (ADN^+/+^) controls, respectively (Liu et al., 2012). Hence, generation of WT and ADN-KO F1 offspring in the same litter requires the genotype of both breeders to be ADN^+/–^, and yet the probability for the concurrent birth of a male WT control and a male ADN-KO mouse by a breeding pair can be fairly low. To deal with this issue, ADN-KO mice and WT controls of the same genetic background were obtained by crossing the corresponding homozygous breeders. While this scheme has been adopted by numerous studies involving this ADN-KO strain, such a practice may not necessarily eliminate environmental factors which potentially add to the genetics ones (e.g., maternal behavior, litter size) and subsequently interfere with the outcomes of experiments. Thus, enlarging the breeding scale or establishing alternative ADN-null strains which allow the concurrence of both ADN-KO and WT mice in a single litter with a greater probability will be more desired in future studies. Furthermore, the turnover of neurotrophic factors is known to be active, so the end-point measurements may not reflect theirs dynamics during the period of more than 3 weeks.

In summary, our results suggest that ADN potentially mediates physical exercise-exerted effects on reducing depression-like behaviors in stressed mice by promoting hippocampal neurogenesis and modulating dendritic plasticity of neurons in the DG. The increased phosphorylation of AMPK following hippocampal ADN elevation may be the enabling step for exercise to enhance NPC proliferation and subsequent neurogenesis. Considering that such a proneurogenic mechanism by ADN does not seem to directly involve other well-known neurotrophic factors, modulation of ADN signaling pathway may accordingly be a novel strategy for treating neuropsychiatric diseases involving stress-elicited impairments of hippocampal neurogenesis and dendritic plasticity. Whether the ADN level could serve as a laboratory index reflecting depression severity and help formulate exercise interventions for specific patients warrants future investigation.

## Data Availability Statement

The datasets generated for this study are available on request to the corresponding author.

## Ethics Statement

The animal study was reviewed and approved by the Animal Ethics Committee of Jinan University.

## Author Contributions

AL, K-FS, and AX conceived and designed the experiments and contributed reagents, materials, and analysis tools. PW, YL, KC, S-YY, KK-YC, and AL performed the experiments and analyzed the data. PW, KC, XS, and AL wrote the manuscript. All authors contributed to the article and approved the submitted version.

## Conflict of Interest

The authors declare that the research was conducted in the absence of any commercial or financial relationships that could be construed as a potential conflict of interest.
