# Supplementary material for: Potential Involvement of Adiponectin Signaling in Regulating Physical Exercise-Elicited Hippocampal Neurogenesis and Dendritic Morphology in Stressed Mice
Source: Front Cell Neurosci. 2020 Jul 3;14:189. doi: 10.3389/fncel.2020.00189 (PMC7381385; doi:10.3389/fncel.2020.00189)
Supplement: Supplementary file 1 [file Data_Sheet_1.pdf]

Supplementary Information

1. Supplemental Tables

Supplemental Table 1. Proprietary Taqman probes for real-time PCR detection from Applied Biosystems Inc.

Target	Assay ID of FAM-labeled MGB Probe
Actb	Mm00607939_s1
AdipoR1	Mm01291334_mH
AdipoR2	Mm01184032_m1
Appl1	Mm00507526_m1

Actb: β-actin; AdipoR1: adiponectin receptor 1; AdipoR2: adiponectin receptor 2; Appl1: adaptor protein containing PH domain, PTB domain and leucine zipper motif 1

Supplemental Table 2. Summary of statistical results by ANOVA

Figure No.	Test Used	n			
1B. Serum COR	Two-way ANOVA	10-11 per group			
**ANOVA Table-1**	**Sum of Squares**	**DF**	**Means of Squares**	**F (DFn, DFd)**	**P value**
Interaction	8.443	1	8.443	F (1, 38) = 0.003204	P=0.9552
Genotype	747.6	1	747.6	F (1, 38) = 0.2837	P=0.5974
Drug	212213	1	212213	F (1, 38) = 80.53	P<0.0001
Residual	100134	38	2635		
**Multiple Comparisons Turkey's multiple comparisons test-1**	**Mean Diff**	**95% CI of diff.**	**Significant?**	**Adjusted P value**	
WVN vs. WCN	-141.4	-201.7 to -81.17	Yes	<0.0001	
KVN vs. KCN	-143.2	-203.5 to -82.97	Yes	<0.0001	
**ANOVA Table-2**	**Sum of Squares**	**DF**	**Means of Squares**	**F (DFn, DFd)**	**P value**
Interaction	2522	1	2522	F (1, 36) = 0.6181	P=0.4369
Genotype	693.9	1	693.9	F (1, 36) = 0.1701	P=0.6825
Exercise	492.8	1	492.8	F (1, 36) = 0.1208	P=0.7302
Residual	146879	36	4080		
**Multiple Comparisons Turkey's multiple comparisons test-2**	**Mean Diff**	**95% CI of diff.**	**Significant?**	**Adjusted P value**	
WCN vs. WCR	8.86	-68.07 to 85.79	No	0.9895	
KCN vs. KCR	-22.9	-99.83 to 54.03	No	0.8532	

Figure No.	Test Used	n			
1C. Sucrose preference test	Two-way ANOVA	8-18 per group			
**ANOVA Table-1**	**Sum of Squares**	**DF**	**Means of Squares**	**F (DFn, DFd)**	**P value**
Interaction	79.92	1	79.92	F (1, 51) = 1.923	P=0.1715
Genotype	57.65	1	57.65	F (1, 51) = 1.387	P=0.2443
Drug	8881	1	8881	F (1, 51) = 213.7	P<0.0001
Residual	2119	51	41.55		
**Multiple Comparisons Turkey's multiple comparisons test-1**	**Mean Diff**	**95% CI of diff.**	**Significant?**	**Adjusted P value**	
WVN vs. WCN	23.99	17.37 to 30.62	Yes	<0.0001	
KVN vs. KCN	29.02	22.03 to 36.01	Yes	<0.0001	
**ANOVA Table-2**	**Sum of Squares**	**DF**	**Means of Squares**	**F (DFn, DFd)**	**P value**
Interaction	296.5	1	296.5	F (1, 38) = 4.803	P=0.0346
Genotype	339.3	1	339.3	F (1, 38) = 5.497	P=0.0244
Exercise	297.6	1	297.6	F (1, 38) = 4.821	P=0.0343
Residual	2346	38	61.73		
**Multiple Comparisons Turkey's multiple comparisons test-2**	**Mean Diff**	**95% CI of diff.**	**Significant?**	**Adjusted P value**	
WCN vs. WCR	-10.88	-20.69 to -1.072	Yes	0.0247	
KCN vs. KCR	-0.01016	-9.028 to 9.008	No	>0.9999	
WCN vs. KCN	0.379	-9.108 to 9.866	No	0.9995	
WCR vs. KCR	11.25	1.894 to 20.60	Yes	0.0131	

Figure No.	Test Used	n			
1D. Force swim test	Two-way ANOVA	13-22 per group			
**ANOVA Table-1**	**Sum of Squares**	**DF**	**Means of Squares**	**F (DFn, DFd)**	**P value**
Interaction	105.9	1	105.9	F (1, 64) = 0.1086	P=0.7429
Genotype	288.6	1	288.6	F (1, 64) = 0.2959	P=0.5884
Drug	77292	1	77292	F (1, 64) = 79.26	P<0.0001
Residual	62414	64	975.2		
**Multiple Comparisons Turkey's multiple comparisons test-1**	**Mean Diff**	**95% CI of diff.**	**Significant?**	**Adjusted P value**	
WVN vs. WCN	-71.16	-97.76 to -44.56	Yes	<0.0001	
KVN vs. KCN	-66.08	-96.84 to -35.32	Yes	<0.0001	
**ANOVA Table-2**	**Sum of Squares**	**DF**	**Means of Squares**	**F (DFn, DFd)**	**P value**
Interaction	4704	1	4704	F (1, 56) = 4.643	P=0.0355
Genotype	5615	1	5615	F (1, 56) = 5.542	P=0.0221
Exercise	10767	1	10767	F (1, 56) = 10.63	P=0.0019
Residual	56730	56	1013		
**Multiple Comparisons Turkey's multiple comparisons test-2**	**Mean Diff**	**95% CI of diff.**	**Significant?**	**Adjusted P value**	
WCN vs. WCR	44.9	15.99 to 73.81	Yes	0.0007	
KCN vs. KCR	9.165	-23.89 to 42.22	No	0.8829	
WCN vs. KCN	-1.653	-32.70 to 29.40	No	0.999	
WCR vs. KCR	-37.39	-68.44 to -6.338	Yes	0.0122	

Figure No.	Test Used	n			
1E. Tail suspension test	Two-way ANOVA	8-21 per group			
**ANOVA Table-1**	**Sum of Squares**	**DF**	**Means of Squares**	**F (DFn, DFd)**	**P value**
Interaction	0.1153	1	0.1153	F (1, 62) = 0.0001856	P=0.9892
Genotype	1066	1	1066	F (1, 62) = 1.717	P=0.1949
Drug	74255	1	74255	F (1, 62) = 119.6	P<0.0001
Residual	38508	62	621.1		
**Multiple Comparisons Turkey's multiple comparisons test-1**	**Mean Diff**	**95% CI of diff.**	**Significant?**	**Adjusted P value**	
WVN vs. WCN	-67.97	-90.21 to -45.72	Yes	<0.0001	
KVN vs. KCN	-67.8	-91.88 to -43.72	Yes	<0.0001	
**ANOVA Table-2**	**Sum of Squares**	**DF**	**Means of Squares**	**F (DFn, DFd)**	**P value**
Interaction	6892	1	6892	F (1, 50) = 13.66	P=0.0005
Genotype	2921	1	2921	F (1, 50) = 5.790	P=0.0199
Drug	4379	1	4379	F (1, 50) = 8.680	P=0.0049
Residual	25223	50	504.5		
**Multiple Comparisons Turkey's multiple comparisons test-2**	**Mean Diff**	**95% CI of diff.**	**Significant?**	**Adjusted P value**	
WCN vs. WCR	42.32	21.18 to 63.47	Yes	<0.0001	
KCN vs. KCR	-4.778	-31.23 to 21.68	No	0.9632	
WCN vs. KCN	8.219	-13.96 to 30.40	No	0.7587	
WCR vs. KCR	-38.88	-64.47 to -13.29	Yes	0.001	

Figure No.	Test Used	n			
1F Hippocampal ADN	Two-way ANOVA	8 per group			
**ANOVA Table-1**	**Sum of Squares**	**DF**	**Means of Squares**	**F (DFn, DFd)**	**P value**
Interaction	0.001724	1	0.001724	F (1, 39) = 0.03608	P=0.8503
Genotype	44.3	1	44.3	F (1, 39) = 927.2	P<0.0001
Drug	0.00157	1	0.00157	F (1, 39) = 0.03286	P=0.8571
Residual	1.863	39	0.04777		
**Multiple Comparisons Turkey's multiple comparisons test-1**	**Mean Diff**	**95% CI of diff.**	**Significant?**	**Adjusted P value**	
WVN vs. WCN	0.026	-0.1957 to 0.2477	No	0.989	
**ANOVA Table-2**	**Sum of Squares**	**DF**	**Means of Squares**	**F (DFn, DFd)**	**P value**
Interaction	0.8518	1	0.8518	F (1, 38) = 13.99	P=0.0006
Genotype	57.54	1	57.54	F (1, 38) = 944.7	P<0.0001
Exercise	0.8259	1	0.8259	F (1, 38) = 13.56	P=0.0007
Residual	2.314	38	0.06091		
**Multiple Comparisons Turkey's multiple comparisons test-2**	**Mean Diff**	**95% CI of diff.**	**Significant?**	**Adjusted P value**	
WCN vs. WCR	-0.5827	-0.8435 to -0.3219	Yes	<0.0001	

Figure No.	Test Used	n			
1G Serum ADN	Two-way ANOVA	8 per group			
**ANOVA Table-1**	**Sum of Squares**	**DF**	**Means of Squares**	**F (DFn, DFd)**	**P value**
Interaction	1.529	1	1.529	F (1, 28) = 0.8765	P=0.3572
Genotype	1003	1	1003	F (1, 28) = 575.0	P<0.0001
Drug	1.519	1	1.519	F (1, 28) = 0.8710	P=0.3587
Residual	48.83	28	1.744		
**Multiple Comparisons Turkey's multiple comparisons test-1**	**Mean Diff**	**95% CI of diff.**	**Significant?**	**Adjusted P value**	
WVN vs. WCN	0.8729	-0.9300 to 2.676	No	0.5572	
**ANOVA Table-2**	**Sum of Squares**	**DF**	**Means of Squares**	**F (DFn, DFd)**	**P value**
Interaction	99.38	1	99.38	F (1, 28) = 11.17	P=0.0024
Genotype	1632	1	1632	F (1, 28) = 183.5	P<0.0001
Exercise	99.07	1	99.07	F (1, 28) = 11.14	P=0.0024
Residual	249.1	28	8.896		
**Multiple Comparisons Turkey's multiple comparisons test-2**	**Mean Diff**	**95% CI of diff.**	**Significant?**	**Adjusted P value**	
WCN vs. WCR	-7.044	-11.12 to -2.972	Yes	0.0003	

Figure No.	Test Used	n			
2A. BrdU	Two-way ANOVA	11-12 per group			
**ANOVA Table-1**	**Sum of Squares**	**DF**	**Means of Squares**	**F (DFn, DFd)**	**P value**
Interaction	423.2	1	423.2	F (1, 42) = 0.04836	P=0.8270
Genotype	672.7	1	672.7	F (1, 42) = 0.07686	P=0.7830
Drug	674466	1	674466	F (1, 42) = 77.07	P<0.0001
Residual	367555	42	8751		
**Multiple Comparisons Turkey's multiple comparisons test-1**	**Mean Diff**	**95% CI of diff.**	**Significant?**	**Adjusted P value**	
WVN vs. WCN	248.5	144.0 to 352.9	Yes	<0.0001	
KVN vs. KCN	236.3	131.9 to 340.8	Yes	<0.0001	
**ANOVA Table-2**	**Sum of Squares**	**DF**	**Means of Squares**	**F (DFn, DFd)**	**P value**
Interaction	72297	1	72297	F (1, 42) = 6.241	P=0.0165
Genotype	49450	1	49450	F (1, 42) = 4.268	P=0.0450
Exercise	117934	1	117934	F (1, 42) = 10.18	P=0.0027
Residual	486570	42	11585		
**Multiple Comparisons Turkey's multiple comparisons test-2**	**Mean Diff**	**95% CI of diff.**	**Significant?**	**Adjusted P value**	
WCN vs. WCR	-180.7	-300.9 to -60.54	Yes	0.0013	
KCN vs. KCR	-22	-142.2 to 98.18	No	0.961	
WCN vs. KCN	-13.73	-136.5 to 109.0	No	0.9906	
WCR vs. KCR	145	27.46 to 262.5	Yes	0.0103	

Figure No.	Test Used	n			
2B. Ki67	Two-way ANOVA	9-15 per group			
**ANOVA Table-1**	**Sum of Squares**	**DF**	**Means of Squares**	**F (DFn, DFd)**	**P value**
Interaction	258.2	1	258.2	F (1, 42) = 0.03207	P=0.8587
Genotype	514.4	1	514.4	F (1, 42) = 0.06390	P=0.8017
Drug	839491	1	839491	F (1, 42) = 104.3	P<0.0001
Residual	338113	42	8050		
**Multiple Comparisons Turkey's multiple comparisons test-1**	**Mean Diff**	**95% CI of diff.**	**Significant?**	**Adjusted P value**	
WVN vs. WCN	272.2	171.0 to 373.4	Yes	<0.0001	
KVN vs. KCN	281.9	177.9 to 386.0	Yes	<0.0001	
**ANOVA Table-2**	**Sum of Squares**	**DF**	**Means of Squares**	**F (DFn, DFd)**	**P value**
Interaction	179968	1	179968	F (1, 37) = 20.75	P<0.0001
Genotype	174673	1	174673	F (1, 37) = 20.14	P<0.0001
Exercise	173217	1	173217	F (1, 37) = 19.97	P<0.0001
Residual	320866	37	8672		
**Multiple Comparisons Turkey's multiple comparisons test-2**	**Mean Diff**	**95% CI of diff.**	**Significant?**	**Adjusted P value**	
WCN vs. WCR	-267.3	-374.4 to -160.3	Yes	<0.0001	
KCN vs. KCR	2.556	-115.5 to 120.6	No	>0.9999	
WCN vs. KCN	-2	-120.1 to 116.1	No	>0.9999	
WCR vs. KCR	267.9	160.9 to 374.9	Yes	<0.0001	

Figure No.	Test Used	n			
2C. DCX	Two-way ANOVA	9-15 per group			
**ANOVA Table-1**	**Sum of Squares**	**DF**	**Means of Squares**	**F (DFn, DFd)**	**P value**
Interaction	229.3	1	229.3	F (1, 44) = 0.01116	P=0.9163
Genotype	3.22	1	3.22	F (1, 44) = 0.0001567	P=0.9901
Drug	1017871	1	1017871	F (1, 44) = 49.54	P<0.0001
Residual	904027	44	20546		
**Multiple Comparisons Turkey's multiple comparisons test-1**	**Mean Diff**	**95% CI of diff.**	**Significant?**	**Adjusted P value**	
WVN vs. WCN	293.6	132.3 to 455.0	Yes	<0.0001	
KVN vs. KCN	302.6	144.1 to 461.0	Yes	<0.0001	
**ANOVA Table-2**	**Sum of Squares**	**DF**	**Means of Squares**	**F (DFn, DFd)**	**P value**
Interaction	218971	1	218971	F (1, 37) = 27.28	P<0.0001
Genotype	230813	1	230813	F (1, 37) = 28.75	P<0.0001
Exercise	214875	1	214875	F (1, 37) = 26.77	P<0.0001
Residual	297006	37	8027		
**Multiple Comparisons Turkey's multiple comparisons test-2**	**Mean Diff**	**95% CI of diff.**	**Significant?**	**Adjusted P value**	
WCN vs. WCR	-294.2	-398.7 to -189.7	Yes	<0.0001	
KCN vs. KCR	1.389	-109.3 to 112.1	No	>0.9999	
WCN vs. KCN	3.944	-106.8 to 114.7	No	0.9997	
WCR vs. KCR	299.6	195.1 to 404.1	Yes	<0.0001	

Figure No.	Test Used	n			
3C. Total dendritic length	Two-way ANOVA	12-17 neurons/group			
**ANOVA Table-1**	**Sum of Squares**	**DF**	**Means of Squares**	**F (DFn, DFd)**	**P value**
Interaction	4351	1	4351	F (1, 57) = 0.1298	P=0.7200
Genotype	62027	1	62027	F (1, 57) = 1.851	P=0.1791
Drug	2797275	1	2797275	F (1, 57) = 83.46	P<0.0001
Residual	1910534	57	33518		
**Multiple Comparisons Turkey's multiple comparisons test-1**	**Mean Diff**	**95% CI of diff.**	**Significant?**	**Adjusted P value**	
WVN vs. WCN	415.2	230.2 to 600.2	Yes	<0.0001	
KVN vs. KCN	449.3	280.5 to 618.0	Yes	<0.0001	
**ANOVA Table-2**	**Sum of Squares**	**DF**	**Means of Squares**	**F (DFn, DFd)**	**P value**
Interaction	210836	1	210836	F (1, 55) = 6.584	P=0.0130
Genotype	592370	1	592370	F (1, 55) = 18.50	P<0.0001
Exercise	524439	1	524439	F (1, 55) = 16.38	P=0.0002
Residual	1761298	55	32024		
**Multiple Comparisons Turkey's multiple comparisons test-2**	**Mean Diff**	**95% CI of diff.**	**Significant?**	**Adjusted P value**	
WCN vs. WCR	-310.3	-496.8 to -123.8	Yes	0.0002	
KCN vs. KCR	-69.49	-245.0 to 106.0	No	0.866	
WCN vs. KCN	81.41	-105.1 to 267.9	No	0.8052	
WCR vs. KCR	322.2	146.7 to 497.7	Yes	<0.0001	

Figure No.	Test Used	n			
3E. Total number of Intersections	Two-way ANOVA	12-17 neurons/group			
**ANOVA Table-1**	**Sum of Squares**	**DF**	**Means of Squares**	**F (DFn, DFd)**	**P value**
Interaction	131.5	1	131.5	F (1, 57) = 0.4701	P=0.4957
Genotype	244	1	244	F (1, 57) = 0.8724	P=0.3542
Drug	20181	1	20181	F (1, 57) = 72.16	P<0.0001
Residual	15940	57	279.7		
**Multiple Comparisons Turkey's multiple comparisons test-1**	**Mean Diff**	**95% CI of diff.**	**Significant?**	**Adjusted P value**	
WVN vs. WCN	33.75	16.85 to 50.65	Yes	<0.0001	
KVN vs. KCN	39.68	24.26 to 55.09	Yes	<0.0001	
**ANOVA Table-2**	**Sum of Squares**	**DF**	**Means of Squares**	**F (DFn, DFd)**	**P value**
Interaction	1530	1	1530	F (1, 55) = 5.516	P=0.0225
Genotype	4331	1	4331	F (1, 55) = 15.62	P=0.0002
Exercise	3082	1	3082	F (1, 55) = 11.11	P=0.0015
Residual	15255	55	277.4		
**Multiple Comparisons Turkey's multiple comparisons test-2**	**Mean Diff**	**95% CI of diff.**	**Significant?**	**Adjusted P value**	
WCN vs. WCR	-24.81	-42.17 to -7.456	Yes	0.0016	
KCN vs. KCR	-4.3	-20.63 to 12.03	No	0.9792	
WCN vs. KCN	7	-10.36 to 24.36	No	0.8558	
WCR vs. KCR	27.51	11.18 to 43.85	Yes	0.0002	

Figure No.	Test Used	n			
4A. Phospho-p38MAPK	Two-way ANOVA	10 per group			
**ANOVA Table-1**	**Sum of Squares**	**DF**	**Means of Squares**	**F (DFn, DFd)**	**P value**
Interaction	133.1	1	133.1	F (1, 36) = 0.7220	P=0.4011
Genotype	0.144	1	0.144	F (1, 36) = 0.0007812	P=0.9779
Drug	12.21	1	12.21	F (1, 36) = 0.06624	P=0.7984
Residual	6636	36	184.3		
**Multiple Comparisons Turkey's multiple comparisons test-1**	**Mean Diff**	**95% CI of diff.**	**Significant?**	**Adjusted P value**	
WVN vs. WCN	-2.543	-18.90 to 13.81	No	0.9749	
KVN vs. KCN	4.753	-11.60 to 21.11	No	0.8617	
**ANOVA Table-2**	**Sum of Squares**	**DF**	**Means of Squares**	**F (DFn, DFd)**	**P value**
Interaction	6.162	1	6.162	F (1, 36) = 0.03980	P=0.8430
Genotype	207.3	1	207.3	F (1, 36) = 1.339	P=0.2548
Exercise	23.08	1	23.08	F (1, 36) = 0.1491	P=0.7017
Residual	5574	36	154.8		
**Multiple Comparisons Turkey's multiple comparisons test-2**	**Mean Diff**	**95% CI of diff.**	**Significant?**	**Adjusted P value**	
WCN vs. WCR	0.7343	-14.25 to 15.72	No	0.9992	
KCN vs. KCR	2.304	-12.68 to 17.29	No	0.9757	
WCN vs. KCN	3.768	-11.22 to 18.76	No	0.9051	
WCR vs. KCR	5.338	-9.650 to 20.33	No	0.7731	

Figure No.	Test Used	n			
4B. Phospho-AKT	Two-way ANOVA	10 per group			
**ANOVA Table-1**	**Sum of Squares**	**DF**	**Means of Squares**	**F (DFn, DFd)**	**P value**
Interaction	55.46	1	55.46	F (1, 36) = 0.04020	P=0.8422
Genotype	117	1	117	F (1, 36) = 0.08479	P=0.7726
Drug	72.25	1	72.25	F (1, 36) = 0.05236	P=0.8203
Residual	49670	36	1380		
**Multiple Comparisons Turkey's multiple comparisons test-1**	**Mean Diff**	**95% CI of diff.**	**Significant?**	**Adjusted P value**	
WVN vs. WCN	-0.3328	-45.07 to 44.41	No	>0.9999	
KVN vs. KCN	-5.043	-49.78 to 39.70	No	0.9901	
**ANOVA Table-2**	**Sum of Squares**	**DF**	**Means of Squares**	**F (DFn, DFd)**	**P value**
Interaction	417.5	1	417.5	F (1, 36) = 0.3326	P=0.5677
Genotype	291.2	1	291.2	F (1, 36) = 0.2320	P=0.6330
Exercise	84.57	1	84.57	F (1, 36) = 0.06738	P=0.7967
Residual	45181	36	1255		
**Multiple Comparisons Turkey's multiple comparisons test-2**	**Mean Diff**	**95% CI of diff.**	**Significant?**	**Adjusted P value**	
WCN vs. WCR	9.369	-33.30 to 52.04	No	0.934	
KCN vs. KCR	-3.553	-46.22 to 39.12	No	0.9959	
WCN vs. KCN	1.065	-41.60 to 43.73	No	0.9999	
WCR vs. KCR	-11.86	-54.53 to 30.81	No	0.8767	
**Figure No.**	**Test Used**	**n**			
4C. Phospho-AMPK	Two-way ANOVA	10 per group			
**ANOVA Table-1**	**Sum of Squares**	**DF**	**Means of Squares**	**F (DFn, DFd)**	**P value**
Interaction	8.264	1	8.264	F (1, 36) = 0.01076	P=0.9180
Genotype	243	1	243	F (1, 36) = 0.3164	P=0.5773
Drug	2548	1	2548	F (1, 36) = 3.317	P=0.0769
Residual	27656	36	768.2		
**Multiple Comparisons Turkey's multiple comparisons test-1**	**Mean Diff**	**95% CI of diff.**	**Significant?**	**Adjusted P value**	
WVN vs. WCN	-15.05	-48.44 to 18.33	No	0.6219	
KVN vs. KCN	-16.87	-50.25 to 16.51	No	0.5313	
**ANOVA Table-2**	**Sum of Squares**	**DF**	**Means of Squares**	**F (DFn, DFd)**	**P value**
Interaction	9209	1	9209	F (1, 36) = 4.685	P=0.0371
Genotype	11811	1	11811	F (1, 36) = 6.008	P=0.0192
Exercise	12696	1	12696	F (1, 36) = 6.459	P=0.0155
Residual	70765	36	1966		
**Multiple Comparisons Turkey's multiple comparisons test-2**	**Mean Diff**	**95% CI of diff.**	**Significant?**	**Adjusted P value**	
WCN vs. WCR	-65.98	-119.4 to -12.58	Yes	0.0104	
KCN vs. KCR	-5.285	-58.69 to 48.12	No	0.9933	
WCN vs. KCN	64.71	11.31 to 118.1	Yes	0.0123	
WCR vs. KCR	299.6	195.1 to 404.1	Yes	<0.0001	

Figure No.	Test Used	n			
4D. Phospho-Erk	Two-way ANOVA	10 per group			
**ANOVA Table-1**	**Sum of Squares**	**DF**	**Means of Squares**	**F (DFn, DFd)**	**P value**
Interaction	376.9	1	376.9	F (1, 36) = 0.1386	P=0.7119
Genotype	73.99	1	73.99	F (1, 36) = 0.02720	P=0.8699
Drug	672	1	672	F (1, 36) = 0.2471	P=0.6222
Residual	97906	36	2720		
**Multiple Comparisons Turkey's multiple comparisons test-1**	**Mean Diff**	**95% CI of diff.**	**Significant?**	**Adjusted P value**	
WVN vs. WCN	-14.34	-77.15 to 48.48	No	0.9267	
KVN vs. KCN	-2.058	-64.87 to 60.75	No	0.9997	
**ANOVA Table-2**	**Sum of Squares**	**DF**	**Means of Squares**	**F (DFn, DFd)**	**P value**
Interaction	1.888	1	1.888	F (1, 36) = 0.0006156	P=0.9803
Genotype	89.09	1	89.09	F (1, 36) = 0.02904	P=0.8656
Exercise	115.5	1	115.5	F (1, 36) = 0.03766	P=0.8472
Residual	110424	36	3067		
**Multiple Comparisons Turkey's multiple comparisons test-2**	**Mean Diff**	**95% CI of diff.**	**Significant?**	**Adjusted P value**	
WCN vs. WCR	3.833	-62.87 to 70.54	No	0.9987	
KCN vs. KCR	2.964	-63.74 to 69.67	No	0.9994	
WCN vs. KCN	3.419	-63.29 to 70.13	No	0.999	
WCR vs. KCR	2.55	-64.16 to 69.26	No	0.9996	

Figure No.	Test Used	n			
5B. Tuj1	Two-way ANOVA	6 per group			
**ANOVA Table**	**Sum of Squares**	**DF**	**Means of Squares**	**F (DFn, DFd)**	**P value**
Interaction	0.1204	1	0.1204	F (1, 20) = 0.08729	P=0.7707
Genotype	0.02042	1	0.02042	F (1, 20) = 0.01480	P=0.9044
Drug	0.0002667	1	0.0002667	F (1, 20) = 0.0001933	P=0.9890
Residual	27.59	20	1.38		
**Multiple Comparisons Turkey's multiple comparisons test**	**Mean Diff**	**95% CI of diff.**	**Significant?**	**Adjusted P value**	
WN vs. WA	-0.1483	-2.046 to 1.750		0.9962	
KN vs. KA	0.135	-1.763 to 2.033	No	0.9971	
WN vs. KN	-0.2	-2.098 to 1.698	No	0.9908	
WA vs. KA	0.08333	-1.815 to 1.981	No	0.9993	

Figure No.	Test Used	n			
5C. Cell phospho-AMPK	One-way ANOVA	4 per group			
**ANOVA Table**	**Sum of Squares**	**DF**	**Means of Squares**	**F (DFn, DFd)**	**P value**
Treatment	10.62	4	2.656	F (4, 15) = 62.70	P<0.0001
Residual	0.6353	15	0.04236		
Total	11.26	19			
**Multiple Comparisons Turkey's multiple comparisons test**	**Mean Diff**	**95% CI of diff.**	**Significant?**	**Adjusted P value**	
Ctrl vs. ADN	-1.62	-2.069 to -1.170	Yes	<0.0001	
Ctrl vs. COR	-0.06275	-0.5121 to 0.3866	No	0.992	
Ctrl vs. ADN + COR	-1.289	-1.738 to -0.8391	Yes	<0.0001	
ADN vs. COR	1.557	1.108 to 2.006	Yes	<0.0001	
ADN vs. ADN + COR + Cpd C	1.725	1.275 to 2.174	Yes	<0.0001	
ADN + COR vs. ADN + COR + Cpd C	1.393	0.9439 to 1.843	Yes	<0.0001	

Figure No.	Test Used	n			
5D. Cell proliferation	One-way ANOVA	3 per group			
**ANOVA Table**	**Sum of Squares**	**DF**	**Means of Squares**	**F (DFn, DFd)**	**P value**
Treatment	34153	3	11384	F (3, 188) = 175.6	P<0.0001
Residual	12189	188	64.83		
Total	46342	191			
**Multiple Comparisons Turkey's multiple comparisons test**	**Mean Diff**	**95% CI of diff.**	**Significant?**	**Adjusted P value**	
Ctrl vs. COR	31.55	27.29 to 35.81	Yes	<0.0001	
Ctrl vs. ADN + COR	7.158	2.898 to 11.42	Yes	0.0001	
Ctrl vs. ADN + COR + Cpd C	27.7	23.44 to 31.96	Yes	<0.0001	
COR vs. ADN + COR	-24.39	-28.65 to -20.13	Yes	<0.0001	
COR vs. ADN + COR + Cpd C	-3.85	-8.110 to 0.4105	No	0.0922	
ADN + COR vs. ADN + COR + Cpd C	20.54	16.28 to 24.80	Yes	<0.0001	

Figure No.	Test Used	n			
sfig.1A. Total moving distance	Two-way ANOVA	13-22 per group			
**ANOVA table-1**	**SS (Type III)**	**DF**	**MS**	**F (DFn, DFd)**	**P value**
Interaction	58482	1	58482	F (1, 60) = 0.06005	P=0.8073
Genotype	3849463	1	3849463	F (1, 60) = 3.952	P=0.0514
Drug	750053	1	750053	F (1, 60) = 0.7701	P=0.3837
Residual	58436445	60	973941		
**ANOVA table-2**	**SS (Type III)**	**DF**	**MS**	**F (DFn, DFd)**	**P value**
Interaction	326201	1	326201	F (1, 76) = 0.3357	P=0.5640
Genotype	1877695	1	1877695	F (1, 76) = 1.932	P=0.1686
Exercise	1043429	1	1043429	F (1, 76) = 1.074	P=0.3034
Residual	73853556	76	971757		
**Figure No.**	**Test Used**	**n**			
sfig.1B. Mean velocity	Two-way ANOVA	13-22 per group			
**ANOVA table-1**	**SS (Type III)**	**DF**	**MS**	**F (DFn, DFd)**	**P value**
Interaction	0.1527	1	0.1527	F (1, 60) = 0.05621	P=0.8134
Genotype	10.87	1	10.87	F (1, 60) = 4.001	P=0.0500
Drug	1.957	1	1.957	F (1, 60) = 0.7205	P=0.3994
Residual	163	60	2.716		
**ANOVA table-2**	**SS (Type III)**	**DF**	**MS**	**F (DFn, DFd)**	**P value**
Interaction	1.085	1	1.085	F (1, 76) = 0.3977	P=0.5302
Genotype	5.009	1	5.009	F (1, 76) = 1.835	P=0.1795
Exercise	2.83	1	2.83	F (1, 76) = 1.037	P=0.3118
Residual	207.4	76	2.729		
**Figure No.**	**Test Used**	**n**			
sfig.2A. Hippocampal BDNF	Two-way ANOVA	12-14 per group			
**ANOVA table-1**	**SS (Type III)**	**DF**	**MS**	**F (DFn, DFd)**	**P value**
Interaction	29.02	1	29.02	F (1, 47) = 0.06794	P=0.7955
Genotype	15.38	1	15.38	F (1, 47) = 0.03601	P=0.8503
Drug	1088	1	1088	F (1, 47) = 2.548	P=0.1172
Residual	20077	47	427.2		
**ANOVA table-2**	**SS (Type III)**	**DF**	**MS**	**F (DFn, DFd)**	**P value**
Interaction	60.39	1	60.39	F (1, 44) = 0.1376	P=0.7125
Genotype	1.635	1	1.635	F (1, 44) = 0.003725	P=0.9516
Exercise	37.35	1	37.35	F (1, 44) = 0.08507	P=0.7719
Residual	19316	44	439		

Figure No.	Test Used	n			
sfig.2B. Hippocampal IGF	Two-way ANOVA	10 per group			
**ANOVA table-1**	**SS (Type III)**	**DF**	**MS**	**F (DFn, DFd)**	**P value**
Interaction	0.0001122	1	0.0001122	F (1, 36) = 0.02642	P=0.8718
Genotype	0.000003025	1	0.000003025	F (1, 36) = 0.0007122	P=0.9789
Drug	0.000003025	1	0.000003025	F (1, 36) = 0.0007122	P=0.9789
Residual	0.1529	36	0.004247		
**ANOVA table-2**	**SS (Type III)**	**DF**	**MS**	**F (DFn, DFd)**	**P value**
Interaction	0.0000676	1	0.0000676	F (1, 36) = 0.01479	P=0.9039
Genotype	0.0002916	1	0.0002916	F (1, 36) = 0.06378	P=0.8021
Exercise	0.003133	1	0.003133	F (1, 36) = 0.6853	P=0.4132
Residual	0.1646	36	0.004572		

Figure No.	Test Used	n			
sfig.2C. Hippocampal VEGF	Two-way ANOVA	5-8 per group			
**ANOVA table-1**	**SS (Type III)**	**DF**	**MS**	**F (DFn, DFd)**	**P value**
Interaction	421.8	1	421.8	F (1, 21) = 2.105	P=0.1616
Genotype	29.05	1	29.05	F (1, 21) = 0.1450	P=0.7072
Drug	168.5	1	168.5	F (1, 21) = 0.8408	P=0.3696
Residual	4208	21	200.4		
**ANOVA table-2**	**SS (Type III)**	**DF**	**MS**	**F (DFn, DFd)**	**P value**
Interaction	826.6	1	826.6	F (1, 26) = 3.214	P=0.0847
Genotype	0.0274	1	0.0274	F (1, 26) = 0.0001066	P=0.9918
Exercise	50.6	1	50.6	F (1, 26) = 0.1967	P=0.6610
Residual	6687	26	257.2		

Figure No.	Test Used	n			
sfig.2D. Hippocampal NGF	Two-way ANOVA	5-8 per group			
**ANOVA table-1**	**SS (Type III)**	**DF**	**MS**	**F (DFn, DFd)**	**P value**
Interaction	0.0002647	1	0.0002647	F (1, 22) = 2.825e-006	P=0.9987
Genotype	88.23	1	88.23	F (1, 22) = 0.9416	P=0.3424
Drug	50.32	1	50.32	F (1, 22) = 0.5370	P=0.4714
Residual	2062	22	93.7		
**ANOVA table-2**	**SS (Type III)**	**DF**	**MS**	**F (DFn, DFd)**	**P value**
Interaction	138.8	1	138.8	F (1, 27) = 1.444	P=0.2399
Genotype	1.86	1	1.86	F (1, 27) = 0.01936	P=0.8904
Exercise	5.629	1	5.629	F (1, 27) = 0.05860	P=0.8105
Residual	2594	27	96.06		

Figure No.	Test Used	n			
sfig.2E. Serum BDNF	Two-way ANOVA	5-10 per group			
**ANOVA table-1**	**SS (Type III)**	**DF**	**MS**	**F (DFn, DFd)**	**P value**
Interaction	0.01521	1	0.01521	F (1, 23) = 0.05245	P=0.8209
Genotype	0.05906	1	0.05906	F (1, 23) = 0.2037	P=0.6560
Drug	0.009325	1	0.009325	F (1, 23) = 0.03217	P=0.8592
Residual	6.668	23	0.2899		
**ANOVA table-2**	**SS (Type III)**	**DF**	**MS**	**F (DFn, DFd)**	**P value**
Interaction	0.04978	1	0.04978	F (1, 30) = 0.08476	P=0.7730
Genotype	0.1292	1	0.1292	F (1, 30) = 0.2200	P=0.6424
Exercise	0.001318	1	0.001318	F (1, 30) = 0.002245	P=0.9625
Residual	17.62	30	0.5874		

Figure No.	Test Used	n			
sfig.2F. Serum IGF	Two-way ANOVA	5-10 per group			
**ANOVA table-1**	**SS (Type III)**	**DF**	**MS**	**F (DFn, DFd)**	**P value**
Interaction	9.507	1	9.507	F (1, 23) = 0.0008812	P=0.9766
Genotype	6082	1	6082	F (1, 23) = 0.5637	P=0.4604
Drug	6.24	1	6.24	F (1, 23) = 0.0005784	P=0.9810
Residual	248146	23	10789		
**ANOVA table-2**	**SS (Type III)**	**DF**	**MS**	**F (DFn, DFd)**	**P value**
Interaction	2578	1	2578	F (1, 30) = 0.2641	P=0.6111
Genotype	1195	1	1195	F (1, 30) = 0.1225	P=0.7288
Exercise	18733	1	18733	F (1, 30) = 1.919	P=0.1761
Residual	292797	30	9760		

Figure No.	Test Used	n			
sfig.2G. Serum VEGF	Two-way ANOVA	5-10 per group			
**ANOVA table-1**	**SS (Type III)**	**DF**	**MS**	**F (DFn, DFd)**	**P value**
Interaction	7.561	1	7.561	F (1, 23) = 0.2029	P=0.6566
Genotype	32.86	1	32.86	F (1, 23) = 0.8819	P=0.3574
Drug	27.75	1	27.75	F (1, 23) = 0.7446	P=0.3971
Residual	857.1	23	37.27		
**ANOVA table-2**	**SS (Type III)**	**DF**	**MS**	**F (DFn, DFd)**	**P value**
Interaction	10.29	1	10.29	F (1, 30) = 0.3496	P=0.5587
Genotype	0.03639	1	0.03639	F (1, 30) = 0.001236	P=0.9722
Exercise	31.09	1	31.09	F (1, 30) = 1.056	P=0.3123
Residual	883.1	30	29.44		

Figure No.	Test Used	n			
sfig.2H. Serum NGF	Two-way ANOVA	5-10 per group			
**ANOVA table-1**	**SS (Type III)**	**DF**	**MS**	**F (DFn, DFd)**	**P value**
Interaction	13.2	1	13.2	F (1, 23) = 1.943	P=0.1767
Genotype	8.029	1	8.029	F (1, 23) = 1.182	P=0.2883
Drug	3.857	1	3.857	F (1, 23) = 0.5676	P=0.4589
Residual	156.3	23	6.795		
**ANOVA table-2**	**SS (Type III)**	**DF**	**MS**	**F (DFn, DFd)**	**P value**
Interaction	18.64	1	18.64	F (1, 30) = 2.099	P=0.1577
Genotype	9.308	1	9.308	F (1, 30) = 1.048	P=0.3141
Exercise	7.963	1	7.963	F (1, 30) = 0.8966	P=0.3513
Residual	266.4	30	8.881		

Figure No.	Test Used	n			
sfig.3. AdipoR1	Two-way ANOVA	6-7 per group			
**ANOVA table-1**	**SS (Type III)**	**DF**	**MS**	**F (DFn, DFd)**	**P value**
Interaction	0.05443	1	0.05443	F (1, 23) = 0.2703	P=0.6081
Genotype	0.1136	1	0.1136	F (1, 23) = 0.5640	P=0.4603
Drug	0.02419	1	0.02419	F (1, 23) = 0.1201	P=0.7320
Residual	4.631	23	0.2014		
**ANOVA table-2**	**SS (Type III)**	**DF**	**MS**	**F (DFn, DFd)**	**P value**
Interaction	0.01955	1	0.01955	F (1, 22) = 0.1116	P=0.7415
Genotype	0.1759	1	0.1759	F (1, 22) = 1.005	P=0.3271
Exercise	0.007915	1	0.007915	F (1, 22) = 0.04521	P=0.8336
Residual	3.852	22	0.1751		

Figure No.	Test Used	n			
sfig.3. AdipoR2	Two-way ANOVA	6-7 per group			
**ANOVA table-1**	**SS (Type III)**	**DF**	**MS**	**F (DFn, DFd)**	**P value**
Interaction	0.04301	1	0.04301	F (1, 23) = 0.3494	P=0.5602
Genotype	0.04301	1	0.04301	F (1, 23) = 0.3494	P=0.5602
Drug	0.01075	1	0.01075	F (1, 23) = 0.08736	P=0.7702
Residual	2.831	23	0.1231		
**ANOVA table-2**	**SS (Type III)**	**DF**	**MS**	**F (DFn, DFd)**	**P value**
Interaction	0.002585	1	0.002585	F (1, 22) = 0.02532	P=0.8750
Genotype	0.2094	1	0.2094	F (1, 22) = 2.051	P=0.1662
Exercise	0.02326	1	0.02326	F (1, 22) = 0.2279	P=0.6378
Residual	2.246	22	0.1021		

**Figure No.**	**Test Used**	**n**			
sfig.3. Appl1	Two-way ANOVA	6-7 per group			
**ANOVA table-1**	**SS (Type III)**	**DF**	**MS**	**F (DFn, DFd)**	**P value**
Interaction	0.008232	1	0.008232	F (1, 23) = 0.1835	P=0.6724
Genotype	0.02033	1	0.02033	F (1, 23) = 0.4530	P=0.5076
Drug	0.0378	1	0.0378	F (1, 23) = 0.8424	P=0.3682
Residual	1.032	23	0.04487		
**ANOVA table-2**	**SS (Type III)**	**DF**	**MS**	**F (DFn, DFd)**	**P value**
Interaction	0.002585	1	0.002585	F (1, 22) = 0.07711	P=0.7838
Genotype	0.03166	1	0.03166	F (1, 22) = 0.9446	P=0.3417
Exercise	0.002585	1	0.002585	F (1, 22) = 0.07711	P=0.7838
Residual	0.7374	22	0.03352		

**Figure No.**	**Test Used**	**n**			
sfig.5B. Spine density	Two-way ANOVA	9-10 branches/group			
**ANOVA table-1**	**SS (Type III)**	**DF**	**MS**	**F (DFn, DFd)**	**P value**
Interaction	0.001091	1	0.001091	F (1, 35) = 0.05080	P=0.8230
Genotype	0.03798	1	0.03798	F (1, 35) = 1.769	P=0.1922
Drug	0.00006425	1	0.00006425	F (1, 35) = 0.002992	P=0.9567
Residual	0.7516	35	0.02147		
**ANOVA table-2**	**SS (Type III)**	**DF**	**MS**	**F (DFn, DFd)**	**P value**
Interaction	0.0001239	1	0.0001239	F (1, 33) = 0.004488	P=0.9470
Genotype	0.0546	1	0.0546	F (1, 33) = 1.978	P=0.1690
Exercise	0.04321	1	0.04321	F (1, 33) = 1.565	P=0.2197
Residual	0.9111	33	0.02761		

**Figure No.**	**Test Used**	**n**			
sfig.5D. PSD95	Two-way ANOVA	5-8 per group			
**ANOVA table-1**	**SS (Type III)**	**DF**	**MS**	**F (DFn, DFd)**	**P value**
Interaction	0.002791	1	0.002791	F (1, 22) = 0.5441	P=0.4685
Genotype	0.0011	1	0.0011	F (1, 22) = 0.2144	P=0.6479
Drug	0.0138	1	0.0138	F (1, 22) = 2.691	P=0.1152
Residual	0.1128	22	0.005129		
**ANOVA table-2**	**SS (Type III)**	**DF**	**MS**	**F (DFn, DFd)**	**P value**
Interaction	0.01314	1	0.01314	F (1, 24) = 3.811	P=0.0627
Genotype	0.0006579	1	0.0006579	F (1, 24) = 0.1909	P=0.6661
Exercise	0.0009514	1	0.0009514	F (1, 24) = 0.2761	P=0.6041
Residual	0.08272	24	0.003447		

**Figure No.**	**Test Used**	**n**			
sfig.5E. GAP43	Two-way ANOVA	5-8 per group			
**ANOVA table-1**	**SS (Type III)**	**DF**	**MS**	**F (DFn, DFd)**	**P value**
Interaction	0.002799	1	0.002799	F (1, 22) = 0.1706	P=0.6836
Genotype	0.02904	1	0.02904	F (1, 22) = 1.769	P=0.1971
Drug	0.03133	1	0.03133	F (1, 22) = 1.909	P=0.1810
Residual	0.3611	22	0.01641		
**ANOVA table-2**	**SS (Type III)**	**DF**	**MS**	**F (DFn, DFd)**	**P value**
Interaction	0.006207	1	0.006207	F (1, 27) = 0.4438	P=0.5109
Genotype	0.02836	1	0.02836	F (1, 27) = 2.028	P=0.1659
Exercise	0.006262	1	0.006262	F (1, 27) = 0.4478	P=0.5091
Residual	0.3776	27	0.01399		

**Figure No.**	**Test Used**	**n**			
sfig.5F. Synaptophysin	Two-way ANOVA	5-8 per group			
**ANOVA table-1**	**SS (Type III)**	**DF**	**MS**	**F (DFn, DFd)**	**P value**
Interaction	0.02364	1	0.02364	F (1, 21) = 4.075	P=0.0565
Genotype	0.0004433	1	0.0004433	F (1, 21) = 0.07641	P=0.7849
Drug	0.01183	1	0.01183	F (1, 21) = 2.039	P=0.1680
Residual	0.1218	21	0.005802		
**ANOVA table-2**	**SS (Type III)**	**DF**	**MS**	**F (DFn, DFd)**	**P value**
Interaction	0.0003019	1	0.0003019	F (1, 24) = 0.04253	P=0.8384
Genotype	0.02866	1	0.02866	F (1, 24) = 4.037	P=0.0559
Exercise	0.008728	1	0.008728	F (1, 24) = 1.229	P=0.2785
Residual	0.1704	24	0.007099		

**Figure No.**	**Test Used**	**n**			
sfig.5G. SNAP25	Two-way ANOVA	5-8 per group			
**ANOVA table-1**	**SS (Type III)**	**DF**	**MS**	**F (DFn, DFd)**	**P value**
Interaction	0.04895	1	0.04895	F (1, 19) = 2.434	P=0.1353
Genotype	0.0231	1	0.0231	F (1, 19) = 1.149	P=0.2973
Drug	0.004044	1	0.004044	F (1, 19) = 0.2010	P=0.6589
Residual	0.3822	19	0.02012		
**ANOVA table-2**	**SS (Type III)**	**DF**	**MS**	**F (DFn, DFd)**	**P value**
Interaction	0.001644	1	0.001644	F (1, 23) = 0.07855	P=0.7818
Genotype	0.001222	1	0.001222	F (1, 23) = 0.05839	P=0.8112
Exercise	0.0008375	1	0.0008375	F (1, 23) = 0.04001	P=0.8432
Residual	0.4814	23	0.02093		

2. Supplementary Figures

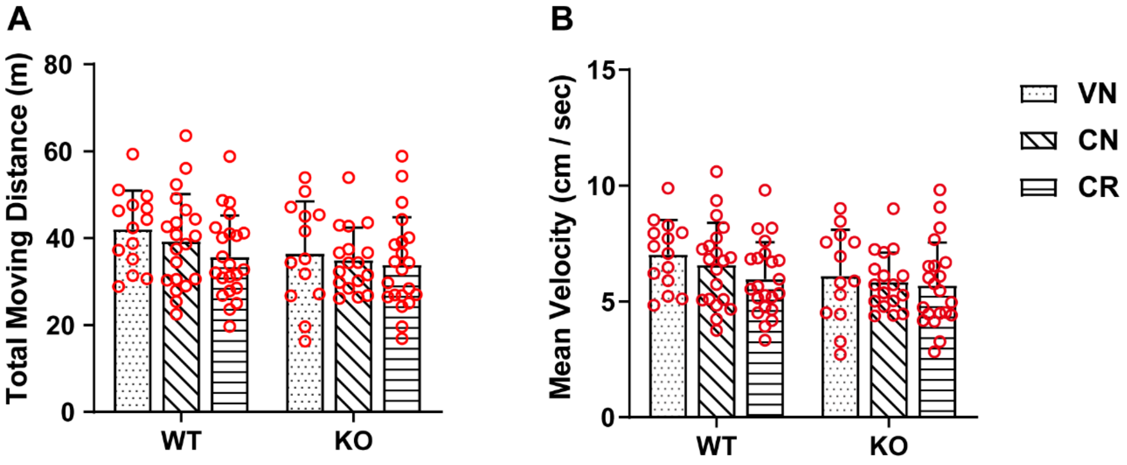

Supplementary Figure 1. The locomotor activity of WT and ADN-KO mice in the open field test

The total moving distance (**A**) and the mean velocity (**B**) assayed by the open field test suggested that COR or physical exercise did not alter the baseline of locomotor activity in WT or ADN-KO mice. n = 13-22 mice/group.

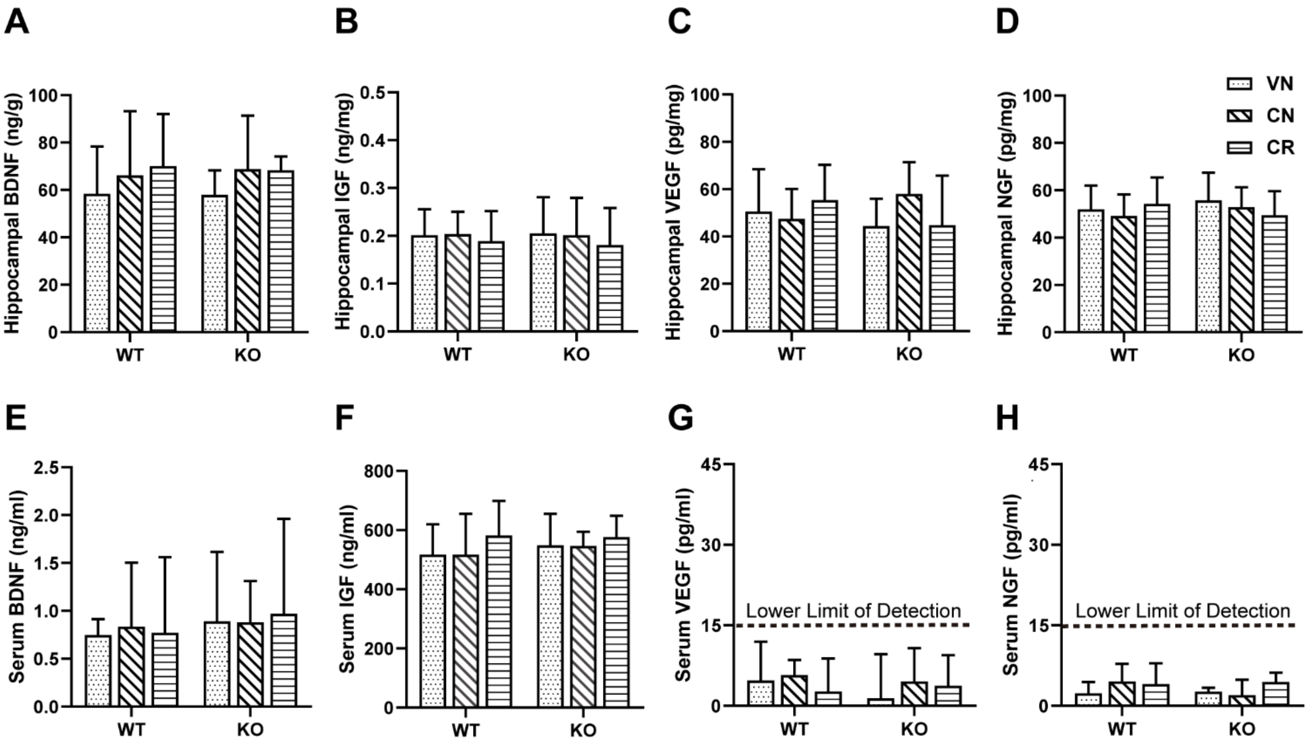

Supplementary Figure 2. Levels of hippocampal and serum neurotrophins after treatments in WT and ADN-KO mice

The hippocampal levels of BDNF (**A**), IGF-1 (**B**), VEGF (**C**) and NGF (**D**) were unaffected by either exercise or ADN KO. Similarly, the serum levels of BDNF (**E**) and IGF-1 (**F**) were also unaltered by either exercise or ADN KO. Concentrations of VEGF (**G**) and NGF (**H**) in the serum were below the lower limit of detection (15 pg/ml). n = 6-8 mice/group. BDNF: brain-derived neurotrophic factor; IGF: insulin-like growth factor 1; VEGF: vascular endothelial growth factor; NGF: nerve growth factor.

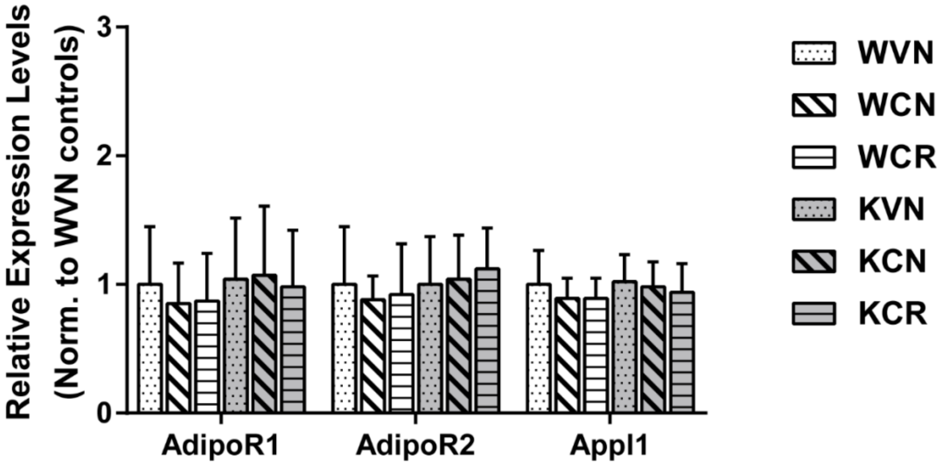

Supplementary Figure 3. Transcriptional expression profiles of AdipoRs and Appl1 in the hippocampus of WT and ADN-KO mice

The results of the real-time PCR quantification confirmed similar expression levels of AdipoR1, AdipoR2 and Appl1 in ADN-KO mice and their WT counterparts. The relative abundances of these three targets were unaltered by exercise or COR-mimicked stress exposure, as evidenced by the comparable levels of the specified targets among all groups. n = 6-8 mice/group. AdipoR1: adiponectin receptor 1; AdipoR2: adiponectin receptor 2; Appl1: adaptor protein containing PH domain, PTB domain and leucine zipper motif 1.

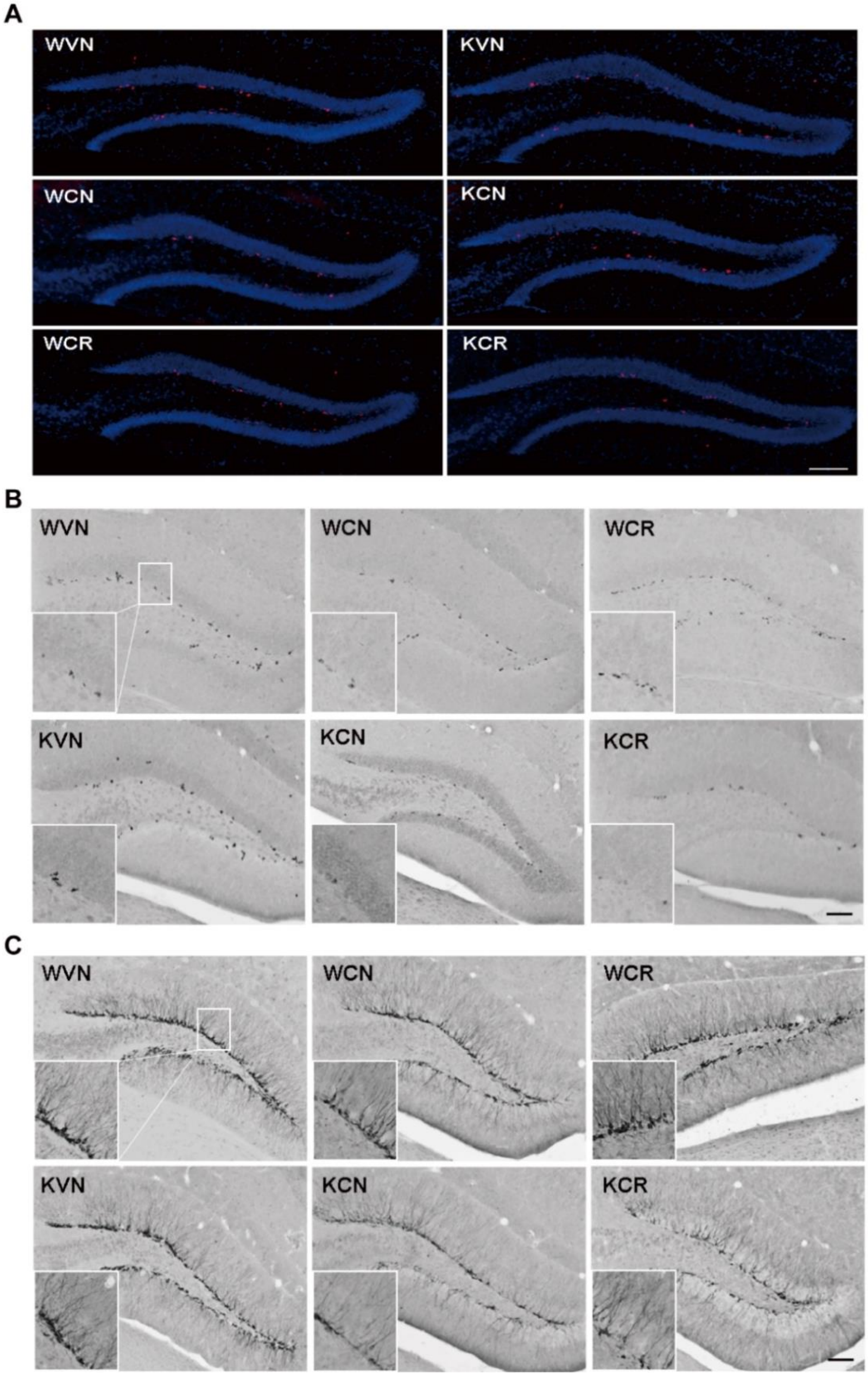

Supplementary Figure 4. Hippocampal neurogenesis in the dentate gyrus

(**A-C**) Representative images showing the newborn (BrdU^{+}; **A**), proliferating (Ki67^{+}; **B**) or immature neuronal (DCX^{+}; **C**) cells in the hippocampal dentate gyrus. Scale bars: 100 μm. BrdU: bromodeoxyuridine; Ki67: cell proliferation-associated nuclear antigen; DCX: doublecortin.

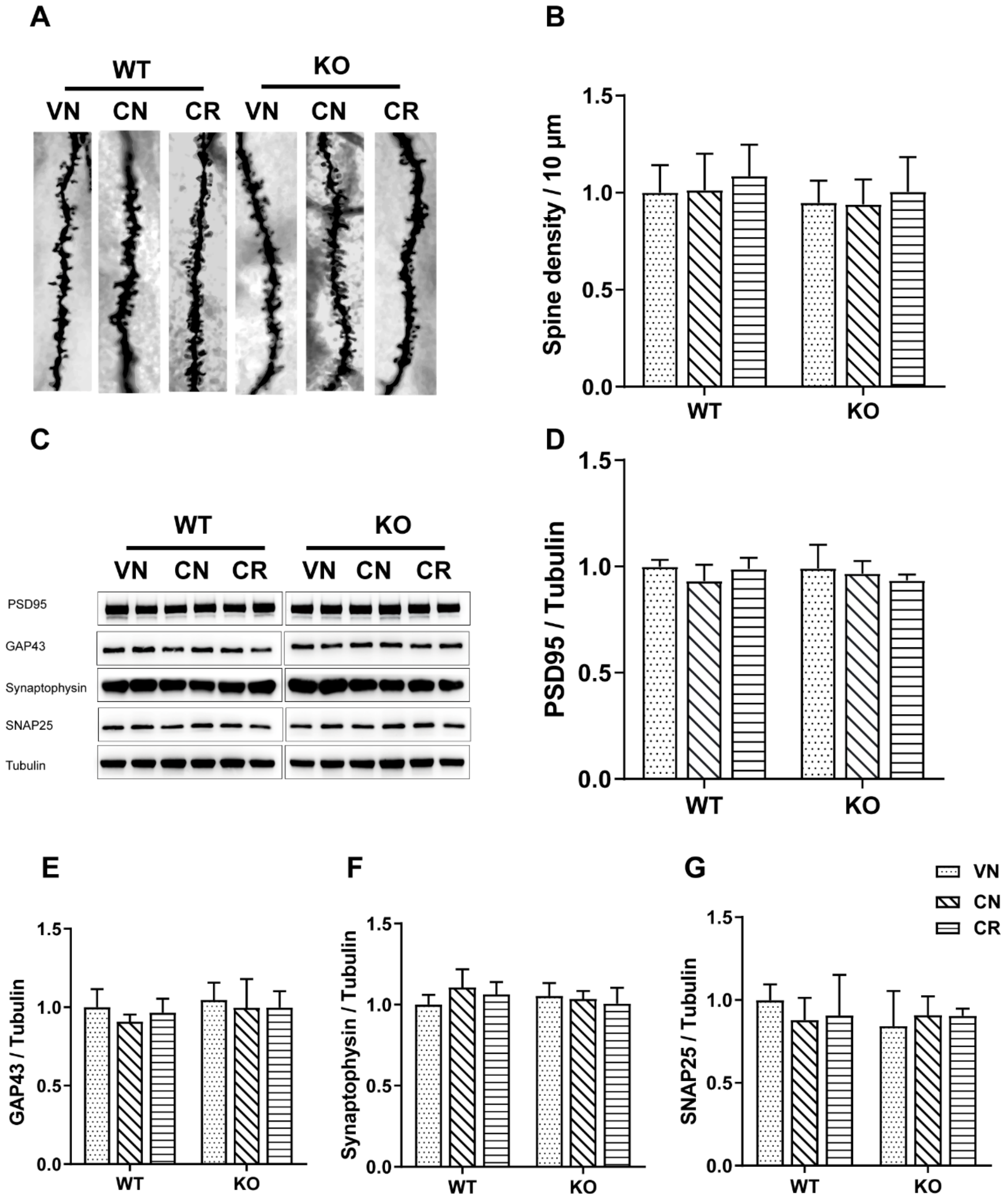

Supplementary Figure 5. Effects of voluntary wheel running on the dendritic spine density of neurons in the DG and hippocampal levels of synaptic proteins of mice under stress

(A) Representative images showing the dendritic spines of neurons in the hippocampal DG. **(B)** COR and voluntary wheel running did not significantly affect the spine density in the hippocampus DG of WT and ADN-KO mice. **(C)** Representative immunoblotting images for PSD95, GAP43, Synaptophysin, SNAP25 and the loading control Tubulin. **(D-G)** Semi-quantitative analyses for PSD95 (**D**), GAP43 (**E**), Synaptophysin (**F**) and SNAP25 (**G**). n = 5-8 mice/group. PSD95: postsynaptic density protein 95; GAP43: growth-associated protein 43; SNAP25: synaptosomal-associated protein 25.

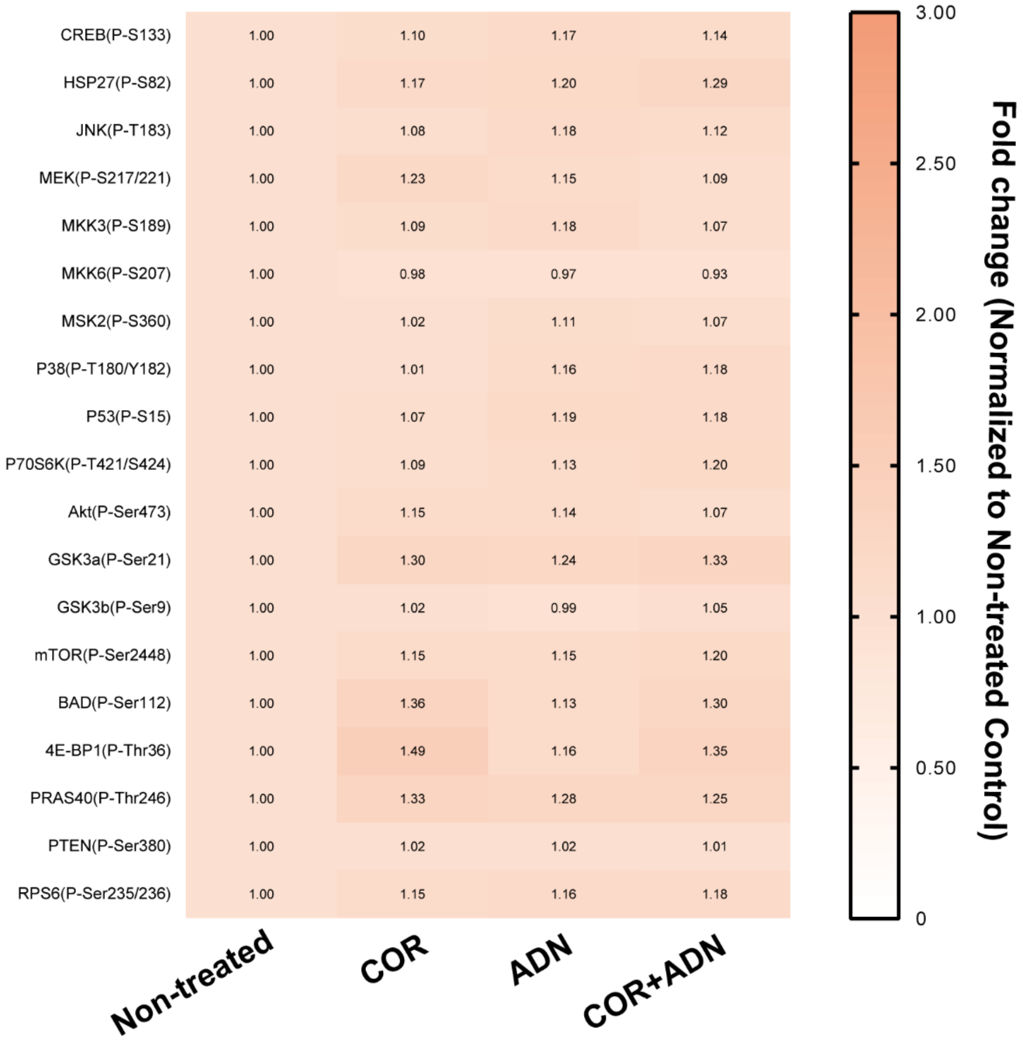

Supplementary Figure 6. Levels of phosphorylated proteins involved in MAPK and AKT pathways
The levels of phosphorylated targets involved in the MAPK pathway and the AKT pathway were determined with the Human/Mouse MAPK Pathway Phosphorylation Array and AKT Pathway Phosphorylation Array C1, respectively. The normalized ratios between 0.8 and 1.5 suggested insignificant changes. Data were averaged from two independent experiments. COR: corticosterone; ADN: adiponectin.
